# The Single-Cell Transcriptomic Analysis of Prefrontal Pyramidal Cells and Interneurons Reveals the Neuronal Expression of Genes Encoding Antimicrobial Peptides and Immune Proteins

**DOI:** 10.3389/fimmu.2021.749433

**Published:** 2021-10-25

**Authors:** Dániel Mittli, Vanda Tukacs, András Micsonai, Lilla Ravasz, József Kardos, Gábor Juhász, Katalin Adrienna Kékesi

**Affiliations:** ^1^ ELTE NAP Neuroimmunology Research Group, Department of Biochemistry, Institute of Biology, ELTE Eötvös Loránd University, Budapest, Hungary; ^2^ Laboratory of Proteomics, Institute of Biology, ELTE Eötvös Loránd University, Budapest, Hungary; ^3^ Clinical Research Units (CRU) Hungary Ltd., Göd, Hungary; ^4^ InnoScience Ltd., Mátranovák, Hungary; ^5^ Department of Physiology and Neurobiology, Institute of Biology, ELTE Eötvös Loránd University, Budapest, Hungary

**Keywords:** antimicrobial peptides, β-defensins, immunomodulatory peptides, neuro-immune interaction, single-cell sequencing, transcriptomics

## Abstract

The investigation of the molecular background of direct communication of neurons and immune cells in the brain is an important issue for understanding physiological and pathological processes in the nervous system. Direct contacts between brain-infiltrating immune cells and neurons, and the neuromodulatory effect of immune cell-derived regulatory peptides are well established. Several aspects of the role of immune and glial cells in the direct neuro-immune communication are also well known; however, there remain many questions regarding the molecular details of signaling from neurons to immune cells. Thus, we report here on the neuronal expression of genes encoding antimicrobial and immunomodulatory peptides, as well as proteins of immune cell-specific activation and communication mechanisms. In the present study, we analyzed the single-cell sequencing data of our previous transcriptomic work, obtained from electrophysiologically identified pyramidal cells and interneurons of the murine prefrontal cortex. We filtered out the genes that may be associated with the direct communication between immune cells and neurons and examined their expression pattern in the neuronal transcriptome. The expression of some of these genes by cortical neurons has not yet been reported. The vast majority of antimicrobial (~53%) and immune cell protein (~94%) transcripts was identified in the transcriptome of the 84 cells, owing to the high sensitivity of ultra-deep sequencing. Several of the antimicrobial and immune process-related protein transcripts showed cell type-specific or enriched expression. Individual neurons transcribed only a fraction of the investigated genes with low copy numbers probably due to the bursting kinetics of gene expression; however, the comparison of our data with available transcriptomic datasets from immune cells and neurons suggests the functional relevance of the reported findings. Accordingly, we propose further experimental and *in silico* studies on the neuronal expression of immune system-related genes and the potential role of the encoded proteins in neuroimmunological processes.

## Introduction

In the last decades, our knowledge about neuro-immune communication progressed and several complex molecular interactions between the immune system and the central nervous system (CNS) have been revealed. It became clear that the main processes of neuro-immune crosstalk in the brain are performed by microglial cells and astrocytes (1). Modulatory effects of immune system-derived cytokines were described in psychiatric and mental illnesses and also in the healthy brain (2, 3). In brain injury or inflammation, the elevated level of cytokines induces behavioral effects as sleepiness, depression, etc. (4). In Alzheimer’s disease, the inflammatory processes are enhanced in the brain in parallel with the upregulation of anti-inflammatory β-defensins (5). However, the neuronal origin of defensins was not established. Defensins are antimicrobial peptides which are part of the innate immune system and have immunomodulatory effects; besides eliminating bacteria, fungi, and viruses, β-defensins were shown to be chemotactic for T cells through C-C chemokine receptor type 6 (CCR6 receptor) (6, 7). Expression of β-defensins and some other antimicrobial peptides has been found in microglial cells and astrocytes (8), but not in neurons.

The action of pro-inflammatory cytokines (e.g., interleukin-1 family, tumor necrosis factor) on neurons is well-known based on the expression of their receptors on nerve cells (9–11). Indeed, neuromodulatory influence of immune cell-derived regulatory peptides is properly established and supports the existence of a communication channel from CNS infiltrating immune cells, microglial cells, and astrocytes to neurons. However, the communication from neurons to adaptive immune cells is less established. Immune cells express the receptors of neurotransmitters (12) and a direct regulatory action of neurotransmitters on T cell maturation is demonstrated (13). T and B cells can enter the CNS under both physiological and pathological conditions (14–16) and some of them can come into close contact with neurons (17, 18). The number of activated T cells increases under brain injury and inflammation (19, 20). The regulation and control of inflammatory processes in the brain may be influenced by the activity of antimicrobial and immunomodulatory peptides (AMPs), similarly to the control of inflammation in peripheral tissues (21). The modulation of T and B cell functions by AMPs is a rapidly growing field in immunology and it is well established that AMPs are expressed by microglial cells and astrocytes as well (22).

Increasing amount of evidence supports that neurons could directly communicate with the cellular components of the innate and/or adaptive immune system; although, it is poorly understood. Direct physical connections of T cells and neurons were uncovered by two-photon microscopy (23). However, the activation of T and B cells by neurons is not completely elucidated in spite of the fact that T and B cells are frequently in active state in the brain. Thus, the question may be raised whether it is a glial cell-mediated indirect action of neurons on adaptive immune cells or there is a direct way of interaction. Therefore, the neuronal expression of proteins - participating in the complex cooperative regulation of T and B cell activation mechanisms and antigen presentation in neurons - is an important issue in understanding the direct bidirectional character of neuro-immune communication, resulting in the complex integration of the immune system and nervous system.

In public databases, as the *in situ* hybridization (ISH) and single-cell sequencing datasets of Allen Institute for Brain Science (24, 25), the expression of many of the genes encoding proteins of the above listed mechanisms is not reported, which may have biological and/or technical reasons. The generally used methods of proteomics and transcriptomics are not able to reveal all low-abundance proteins and their mRNAs, since their sensitivity is not high enough. The protein composition of a cell canonically contains ~20,000 different proteins; in turn, the number of mRNA species transcribed from DNA in a cell should also be ~20,000 (26). In contrast, the average number of detected proteins in a proteomics sample rarely exceeds 3,000 (27) and the number of identified mRNAs in single-cell samples is rarely higher than 2,000 due to the applied sequencing method limited by the number of cells and the concomitant budget requirements. The cellular heterogeneity of the brain tissue evidently does not allow the search for low-abundance proteins. In the last decade a highly sensitive single-cell sequencing method was developed by combination of single-cell harvesting after patch clamping and ultra-deep mRNA sequencing (28). Ultra-deep sequencing allows the detection of extremely low mRNA copy numbers in biological samples, so the results are rich in low-abundance mRNAs (29). In the present work, we analyzed the data of our earlier single-cell sequencing study of prefrontal neurons (30) and, using bioinformatic tools, we identified transcripts encoding proteins of immune cell-specific processes and AMPs. The aim was to provide evidence on the possibility of neuron to adaptive immune cell communication in the brain. *In silico* analysis of the data revealed high transcriptomic coverage of T cell activation, B cell activation, and antigen presentation processes, as well as the expression of several AMP genes in pyramidal cells and fast spiking interneurons of the murine prefrontal cortex. Some of the uncovered mRNAs were unknown in cortical neurons before as established by the comparison of our data with available datasets; however, the transcriptomic similarities found between immune cells, the neurons we sequenced, and other CNS neurons suggest the functional relevance of the neuronal expression of immune system-related genes.

## Materials and Methods

### Cell Harvesting From Acute Brain Slices

The single-cell transcriptomic data analyzed in the present *in silico* study are from our previous work (30; GEO accession number: GSE135060), which includes the detailed methodological description of brain slice preparation, cell harvesting, single-cell sequencing, and data preprocessing. Here, we refer only briefly to these previously performed experimental parts of our work and discuss the bioinformatics analyzes that form the basis of the present study.

We used patch-seq to obtain complex electrophysiological and transcriptomic data from single prefrontal cortical pyramidal (Pyr) cells and fast-spiking (FS) interneurons. This technique is an improvement of the classical whole-cell patch clamp method that allows analyzing the mRNA content of the neuronal cytoplasm harvested by a patch pipette after electrophysiological recordings. Single cortical neurons were harvested from acute brain slices prepared from male C57BL/6N mice (n=73) of age between 27 and 40 days (Innovo Kft., Isaszeg, Hungary). All procedures of animal care and the minimization of the suffering and pain of the animals were done under the ethical rules of Eötvös Loránd University in accordance with the EU Ethical Rules of Using Animals for Research Purposes (2010/63/EU revising Directive 86/609/EEC) and the Hungarian Act of Animal Care and Experimentation (1998, XXVIII).

We used standard brain slice preparation technology, paying special attention to sterile conditions to avoid bacterial contaminations and degradation of mRNAs by RNases. Mice were quickly anesthetized in isoflurane and decapitated. Brains were removed and 300 µm coronal slices were cut by a vibratome (Leica VT1000 S, Leica Biosystems, Wetzlar, Germany) in ice cold artificial cerebrospinal fluid (ACSF). Slices were then incubated in ACSF at room temperature and permanently supplied with carbogen for at least 1 hour before recording and cell harvesting. We pulled 4-10 MΩ resistance patch pipettes just before use by a vertical pipette puller (Model 720, David Kopf Instruments, Tujunga, CA, USA) and filled with sterile, RNase-free intracellular solution (see 30 for the exact composition of the solutions).

Whole-cell patch clamp recordings were performed using a standard *ex vivo* electrophysiological setup built in a Faraday cage and equipped with a DIC microscope (Leica DM6000 FS, Leica Microsystems, Wetzlar, Germany). A Sutter MP-285 micromanipulator (Sutter Instrument, Novato, CA, USA) was used for fine movement of patch pipettes. The whole equipment was placed on a Gibraltar platform and X-Y stage (Burleigh Instruments, New York, NY, USA). During the patch clamp recordings, we applied a step-gradient depolarization protocol in bridge mode. Electrophysiological signals were sampled at 10 kHz, amplified by an AxoClamp 2B amplifier (Axon Instruments, Foster City, CA, USA), and digitized by a CED 1401 MK II (Cambridge Electronic Design, Cambridge, UK) data capture device using CED Signal 4.11 software.

For cell type identification, we used morphological and electrophysiological features. Pyr cells were identified on the basis of their larger, pyramid-like cell body, visible apical dendrite, and regular firing pattern. FS interneurons were recognized based on their small, spherical cell body, characteristically high firing rate, and narrow action potentials. We harvested at most 2 cells from each type from one animal and sequenced altogether 59 Pyr and 25 FS cells.

### Amplification and Sequencing

After whole-cell patch clamp recording, the cytoplasm of the recorded cell was harvested by aspiration into the patch pipette. The mRNA content of the harvested cytoplasm was reverse transcribed into cDNA, amplified through aRNA amplification (31, 32), and libraries were constructed for deep sequencing. Neuronal mRNA content was amplified individually for each cell through three aRNA amplification rounds. ERCC RNA Spike-In controls (Life Technologies, Carlsbad, CA, USA) were added in 1:4,000,000 dilutions to each sample to control for technical variability between samples. During the first amplification round, a synthesized oligo(dT)-T7 primer was annealed to the poly-A tail of the mRNA. cDNA was synthesized using SuperScript III reverse Transcriptase (Invitrogen, Carlsbad, CA, USA), and DNA polymerase I (Invitrogen) following the instructions of the manufacturer. The cDNA served as template during aRNA synthesis using the MEGAscript T7 kit (Invitrogen). For the second and third RNA amplification rounds, aRNA was converted into cDNA using random hexanucleotide primers for the first strand and oligo(dT)-T7 primer for the second strand synthesis. After amplification the aRNA was purified with AGENCOURT RNACLEAN X beads (Beckman Coulter, Brea, CA, USA) and controlled for quality and quantity using Bioanalyzer RNA Picochip and Nanochip (Agilent, Santa Clara, CA, USA).

Libraries were constructed from the aRNA applying the TruSeq Stranded mRNA Library Prep Kit (Illumina, San Diego, CA, USA) without performing the initial fragmentation incubation step. The libraries were quantified using Bioanalyzer DNA1000 chip (Agilent) and sequenced either on HiSeq2500 (producing 100-base paired-end reads), or NextSeq500 (producing 75-base paired-end reads).

### Normalization of Raw Sequencing Data

Because of the low sample volumes, the technical difficulties of cell harvesting, and the differences in the actual molecular state of individual cells, the raw single-cell sequencing data shows high variability in the number of expressed genes per cell and in their copy numbers. Therefore, the normalization of the raw sequencing data is a critical issue in transcriptomic analysis of a heterogeneous neuron population. We believe that after adequate normalization, the sum of normalized copy numbers should correlate positively with the number of recovered transcripts. The sequencing data was first normalized using the DESeq R program package (developed for bulk sequencing experiments), which resulted in a poor correlation. The raw copy number data was then normalized on housekeeping genes. However, this normalization method resulted also in a rather weak correlation. We observed that also these genes were expressed only in subsets of neurons and none of the cells expressed all of the genes encoding housekeeping proteins. This incomplete transcription pattern of housekeeping genes may ensue from technical uncertainties or, more likely, from the bursting kinetics of the mammalian gene transcription. As it is well established, gene expression is performed in bursts (33, 34) and several genes could be in OFF state in the time point of cytoplasm harvesting. We found that several genes are transcribed in large copy numbers but only in a few cells, and probably gene OFF state is responsible for the zero transcription values. Thus, reference genes suitable for normalization should be transcribed in the majority of cells and their copy numbers should be high and stable. Based on these considerations, we searched for reference genes in an unbiased way, without knowing the function of the encoded proteins.

First, we sorted out the 1,000 most frequently expressed genes. To find the most constantly expressed genes, we carried out a “pre-normalization” on the sequencing data using these 1,000 genes as reference (for further details please see 30). Then, from the “pre-normalized” data, we sorted out the 500 most stable genes showing the lowest standard deviation between cells. In the next step we further searched for transcripts that show less than tenfold copy number differences between Pyr and FS cells. The selected 409 genes were used as reference genes for copy number normalization. These genes, showing frequent and stable expression in both types of neurons represent the “equi-phenotype” of the sequenced neuronal transcriptomes. It is important to note, that for the majority of genes, sequencing data showed zero copy numbers in several neurons. The high ratio of zero copy numbers considerably lowers the average transcription values, and the calculation of median for single-cell transcriptomic data could be misleading as well. Thus, the average copy numbers were calculated only on the basis of non-zero values (i.e., based on the data of cells in which the genes were in ON state). The rationale of this approach is that the average of non-zero copy numbers may reliably represent the average transcription level in the gene ON state. Together with the ON state frequency, this estimation can provide a more real picture of single-cell transcriptomics than the overall average or median including zero values (gene OFF states). The selected 409 genes were used to normalize the raw data of the individual cells for transcript copy number analyses. The normalization factor for a cell was the multiplication-factor that provided the lowest root-mean-square deviation on the 409 reference genes compared to the averages of the non-zero values. The remarkable positive correlation (Pearson correlation coefficient: *r* = 0.806) between the number of detected transcripts and the sum of copy numbers indicates that our normalization method can be reliably applied during the preprocessing of single-cell transcriptomic data. The raw sequencing data of Pyr and FS cells were compared on the 409 genes of “equi-phenotype” and on all the genes sequenced. As an average, we detected twice higher amount of mRNA molecules in Pyr cells which may be explained by their larger cell body and higher volume of cytoplasm.

We intended to compare the transcription levels of the investigated genes between FS and Pyr cells to identify cell type-specific or enriched transcripts. However, it was challenging to find an appropriate statistical method to verify the reliability of comparison and reduce false discovery rate, because relatively few cells were sequenced, and the majority of genes were transcribed in only a fraction of cells resulting in a lot of zero copy numbers. Thus, we performed a randomized group comparison analysis to demonstrate the reliability of identifying differentially expressed genes between Pyr and FS cells (for the exact details of the analysis please see 30).

### Investigation of the Expression of Genes Encoding AMPs and Proteins of Immunological Processes

To better understand the possible molecular basics of direct neuro-immune interactions and the immunomodulatory potential of cortical neurons, we analyzed the expression pattern of genes encoding AMPs and proteins playing role in immunological processes (T cell dependent B cell activation, T cell independent B cell activation, CD8+ T cell activation, MHC class I antigen presentation, and MHC class II antigen presentation). The list of AMPs was taken from a recently published study (35), which presents a unified comprehensive human antimicrobial and immunomodulatory protein database constructed by collecting and integrating data from online databases and literature search. Based on this dataset, we searched for AMP transcripts in our normalized sequencing data. The reference list of proteins involved in the studied immunological processes was accessed in the Curated Pathways library of Elsevier Pathway Studio v11.0 (36), and based on this, corresponding mRNAs were searched in the sequencing data of Pyr and FS cells.

For each investigated immunological gene, ON and OFF state frequencies were calculated, i.e. the percentage of neurons expressing and not expressing a particular gene. To reveal transcriptomic differences between Pyr and FS cells, the copy numbers of AMP and immune process protein transcripts were compared and cell type-specific (expressed only in Pyr or FS cells) and enriched (having more than tenfold higher copy numbers in one of the cell types) transcripts were identified. We compared the average copy numbers per cell calculated on the basis of ON states (i.e., non-zero values), as described earlier. The differential distribution of transcripts by cell type may suggest that the neuronal expression of immunological genes is a functionally relevant phenomenon. Before performing statistical comparison or correlation analysis, the normal distribution of data was checked with Shapiro-Wilk test using the Statistics toolbox of OriginPro 8.5 software (OriginLab Corporation, Northampton, MA, USA). Data are presented as average ± standard deviation (SD).

For visualization of transcriptomic data, we built interaction networks of the encoded AMPs and proteins of immune cell processes. Although it is not evident how protein abundances can be inferred from mRNA data, this method may be still suitable for demonstrating the coverage of the studied immunological processes and molecular networks at the transcriptomic level. Interactions of AMPs and proteins from each immunological process were searched with STRING database (37); interactions were excluded below the combined score of 0.7. Then, results were exported to Cytoscape v3.7.2 (38), and protein-protein interaction networks were supplemented with additional interactions from Pathway Studio v11.0. Nodes were colored based on the percentage of neurons in which the corresponding transcripts were detected (ON state frequency). Then, interactions between the AMPs and immunological processes were also searched in STRING; based on their interactions, edges were introduced between AMPs and proteins involved in the examined immune cell processes.

To compare our results to earlier transcriptomic data from the mouse brain, we reviewed the ISH data of Allen Mouse Brain Atlas (24) and the single-cell sequencing data (whole cortex and hippocampus, SMART-seq (2019), Transcriptomics Explorer) of Allen Cell Types Database (25) for the transcripts we have identified. In the ISH data, we searched for genes that showed non-zero raw expression value in any brain structure. Genes with non-zero trimmed mean expression values in at least one cell of the neuronal cell types were searched in the single-cell transcriptomic data.

To further elucidate the possible functional significance of the neuronal expression of immunological genes, we searched the identified AMP and immune process transcripts in online available single-cell sequencing data. The following datasets were reviewed, and the ON state frequencies of the investigated genes were calculated: GSE100337 (39), GSE108989 (40), GSE119373 (41), GSE120575 (42), GSE124675 (43), GSE126030 (44), GSE98638 (45), and GSE75386 (46). These datasets were obtained from human immune cells, with the exception of the latter, which was derived from mouse hippocampal neurons. No quantitative analysis or statistical comparison was performed due to the different experimental and data analysis methods.

## Results

The raw sequencing data of the 84 prefrontal cortical (PFC) neurons contained more than 19,000 transcripts, which is comparable to the estimated number of proteins in a mammalian cell (26). However, the number of different transcripts found in single neurons was between 498 and 9,680 (on average 3,420). Thus, each neuron contained a fraction of all identified transcripts at the time of cell harvesting, as due to the bursting kinetics of transcription, genes may often be in OFF state even for longer periods (47). Using published databases of AMPs and immune cell protein networks as reference lists, we were able to identify transcripts encoding AMPs with antimicrobial and immunomodulatory functions, as well as proteins of antigen presentation and T and B cell activation pathways. The list of AMP transcripts identified in PFC neurons together with the ON state frequencies and average normalized copy numbers is presented in **Supplementary Table 1**. In **Supplementary Table 2**, we show the reference list of transcripts encoding the proteins of different immune cell processes, containing the transcriptomic data as well.

### Examination of the Neuronal Transcriptomic Pattern of Genes Encoding AMPs

**Figure 1:**
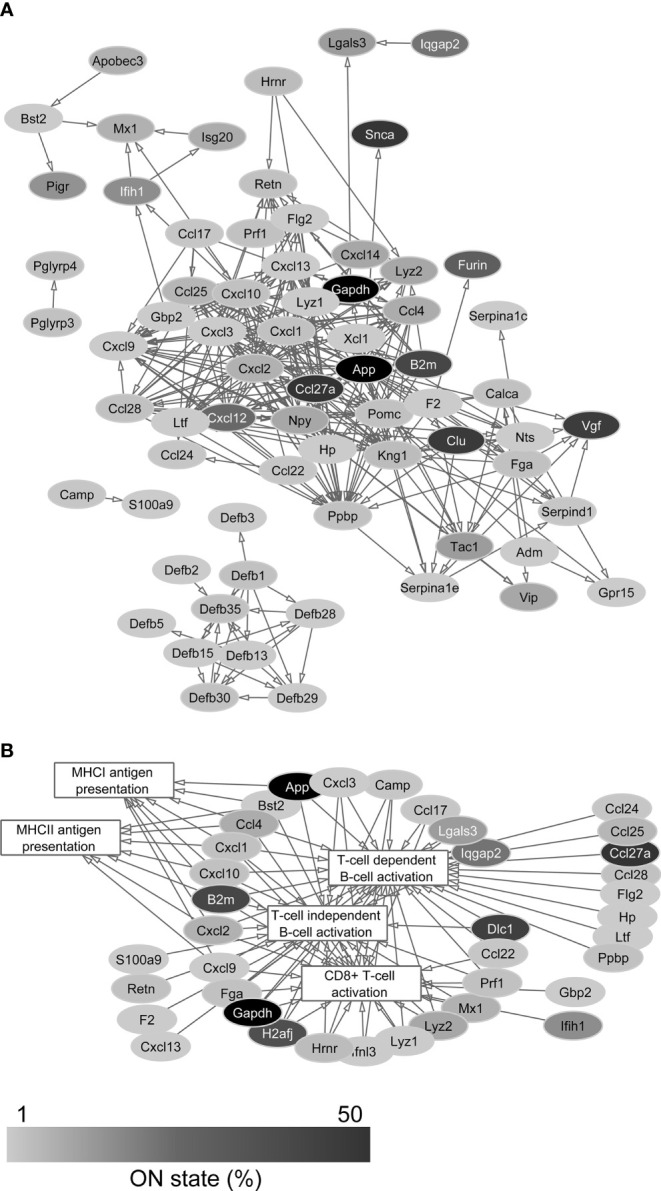
Protein-protein interaction networks of AMPs **(A)** and their association with immune cell processes **(B)**. The large number of connections suggests the immunomodulatory function of AMPs besides their antimicrobial role. Gray scale shows the percentage of neurons that expresses the transcript of each protein in the single-cell sequencing data (ON state frequency).

**Figure 2:**
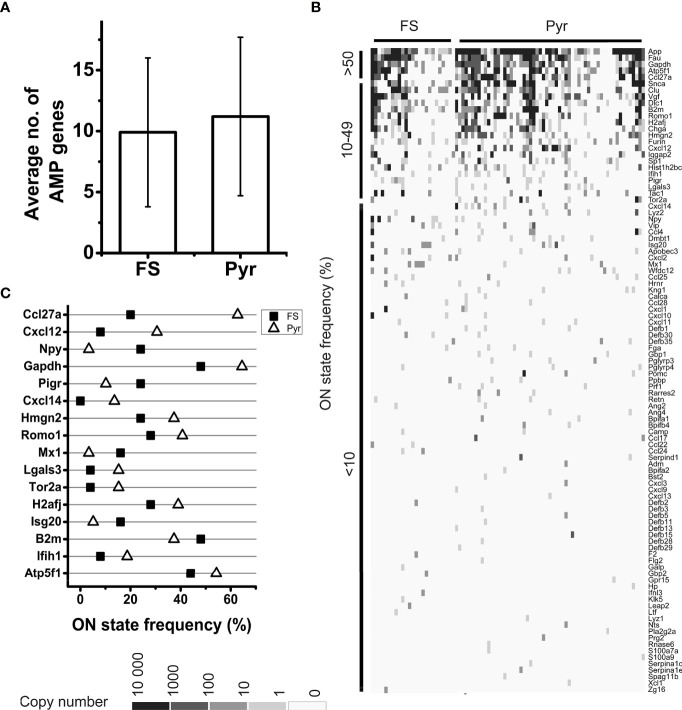
Analysis of AMP mRNA expression by FS and Pyr cells. Bar plot shows the average number of expressed AMP genes in FS and Pyr cells, error bars represent SD **(A)**. Heatmap of AMP gene expression in single neurons; genes are ordered based on their ON state frequencies; gray scale shows the copy number of mRNAs in each cell **(B)**. ON state frequencies of genes in FS and Pyr cells are shown; only those genes are listed, which have at least 10% difference in their ON state frequency between the two cell types **(C)**.

The list of human AMPs published by Kumar et al. contains 186 peptides and proteins with known antimicrobial and/or immunomodulatory effect (35). One hundred of them (53.8%) were found in the transcriptomes of the sequenced PFC neurons (**Supplementary Table 1**). The protein network of AMPs encoded by the identified transcripts could be composed from protein-protein interaction databases using STRING and Cytoscape platforms. **Figure 1A** shows the functional network of AMPs and indicates the ON state frequency of their genes in our sequencing data. Based on network analysis, the AMPs encoded by the identified transcripts are strongly linked to the investigated immunological processes (**Figure 1B**) demonstrating their role in the modulation of immune cell functions. The individual cells expressed averagely 10.8 ± 6.4 different transcripts encoding AMPs; in this regard we found no significant differences between Pyr (11.2 ± 6.5) and FS (9.9 ± 6.1) cells (*P* = 0.39, two-sample *t*-test) (**Figure 2A**).

The ON state frequency of the identified AMP transcripts was rather low (**Figures 1**, **2B**). Out of the 100 genes encoding AMPs only 5 (*App*, *Atp5f1*, *Ccl27a*, *Fau*, and *Gapdh*) were expressed in at least 50% of the neurons, 19 (e.g., *Cxcl12*, *Furin*, *Ifih1*, *Pigr*, and *Romo1*) were expressed in 10-49% of the cells, and 76 (e.g., several β-defensins, CC and CXC motif chemokines) were transcribed in less than 10% of the cells (**Supplementary Table 1** and **Figure 2B**). The average ON state frequency of AMP genes is similar in Pyr (11.2±17.6%) and FS (9.9±15.7%) cells. For the majority of transcripts, it can be observed that higher ON state frequencies are significantly (*P* = 2.6*10^-13^) associated with higher average copy numbers (Spearman correlation coefficient: *r_S_* = 0.65, **Supplementary Figure 1**), suggesting that the detection of AMP transcripts does not result from methodological bias or random gene expression noise, but possibly from regulated biological processes. The average normalized copy numbers of AMP transcripts ranged from 0.1 to 1063.9 (on average 75.3, see **Supplementary Table 1** for each gene). For comparison, we present here the expression data of some genes encoding proteins with well-known neuronal functions: *Syt4* (synaptotagmin-4) was expressed by 66.7% of the neurons with 519.3 average copy number, *Scn3a* (encoding a sodium channel subunit) was expressed by 51.2% of the neurons with 335.6 average copy number, *Gria1* (encoding a glutamate receptor subunit) was expressed by 67.9% of the cells with 864.5 average copy number, and *Dlg4* (encoding postsynaptic density protein 95 (PSD-95), a postsynaptic scaffolding protein) was expressed by 44.1% of the cells with 149.9 average copy number.

**Table 1** shows the distribution by cell type of the 100 identified AMP transcripts. Thirty-four of them were evenly expressed in both cell types (i.e., showing less than tenfold copy number differences). Among these there are some that, in addition to its presumable antimicrobial and/or immunomodulatory role, also have more general cellular functions (e.g., *Atp5f1*, *Gapdh*, *H2aj*, and *Hist1h2bc*). Fifteen of the AMP transcripts showed cell type-enriched expression (i.e., having more than tenfold higher copy numbers in one of the cell types). These encode mainly proteins with well-documented antimicrobial or other immunological functions (e.g., *Lyz2* and *Cxcl12* enriched in Pyr cells or *Mx1* and *Ccl4* enriched in FS cells). Interestingly, 51 AMP genes were expressed in a cell type-specific manner. Forty of them were identified only in Pyr cells (e.g., 8 of the 11 β-defensin genes found in the neurons, *Lyz1*, *Prf1*, *Cxcl14*, and *Rnase6*), and the remaining 11 were transcribed only in FS cells (e.g., *Ccl22*, *Galp*, and *Ifnl3*). *Cxcl12*, *Mx1*, and *Cxcl14* also showed high difference in their ON state frequencies between cell types (**Figure 2C**). The ON state frequency of *Cxcl12* was 8.0% and 30.5% in FS and Pyr cells, respectively; *Mx1* was detected in 16.0% of FS and 3.4% of Pyr cells. Finally, *Cxcl14* was expressed in 13.6% of Pyr cells and was not detected in FS cells.

**Table 1:** The distribution of AMP transcripts in the investigated Pyr and FS cells.

Genes expressed in Pyr and FS cells			Genes expressed only in Pyr or FS cells	
Less than tenfold differences in copy numbers	More than tenfold higher copy numbers in Pyr cells	More than tenfold higher copy numbers in FS cells	Expressed only in Pyr cells	Expressed only in FS cells
*Ang2*, *App*, *Atp5f1*, *B2m*, *Calca*, *Camp*, *Ccl25*, *Ccl27a*, *Ccl28*, *Chga*, *Clu*, *Cxcl11*, *Defb35*, *Dmbt1*, *Fau*, *Fga*, *Furin*, *Gapdh*, *H2aj*, *Hist1h2bc*, *Hmgn2*, *Hrnr*, *Ifih1*, *Iqgap2*, *Isg20*, *Lgals3*, *Pglyrp4*, *Pigr*, *Ppbp*, *Retn*, *Sp1*, *Vgf*, *Vip*, *Wfdc12*	*Cxcl12*, *Dlc1*, *Lyz2*, *Romo1*, *Snca*, *Tor2a*	*Apobec3*, *Ccl4*, *Cxcl1*, *Cxcl2*, *Cxcl10*, *Defb30*, *Mx1*, *Npy*, *Tac1*	*Adm*, *Ang4*, *Bpifa1*, *Bpifa2*, *Bpifb4*, *Bst2*, *Ccl17*, *Cxcl3*, *Cxcl9*, *Cxcl13*, *Cxcl14*, *Defb1*, *Defb3*, *Defb5*, *Defb11*, *Defb13*, *Defb15*, *Defb28*, *Defb29*, *Flg2*, *Gbp1*, *Gpr15*, *Hp*, *Kng1*, *Lyz1*, *Nts*, *Pglyrp3*, *Pla2g2a*, *Pomc*, *Prf1*, *Prg2*, *Rarres2*, *Rnase6*, *S100a7a*, *S100a9*, *Serpina1c*, *Serpina1e*, *Serpind1*, *Spag11b*, *Xcl1*	*Ccl22*, *Ccl24*, *Defb2*, *F2*, *Galp*, *Gbp2*, *Ifnl3*, *Klk5*, *Leap2*, *Ltf*, *Zg16*
**34.0%**	**6.0%**	**9.0%**	**40.0%**	**11.0%**

Several genes were expressed in both neuron types with similar copy numbers. However, the majority of AMP genes showed cell type-specific or enriched expression. In particular, we found many transcripts that could be identified only in Pyr cells. We compared the average copy numbers calculated on the basis of ON state cells (i.e., non-zero values), as described in the Materials and Methods.

Bold values indicate the percentage of genes belonging to the group.

Thus, based on the sequencing data, it can be established that the Pyr and FS cells of the murine PFC are able to express several AMP genes. The majority of transcripts could be identified in few cells (low ON state frequency) with low average copy numbers; however, several of them show cell type-specific or enriched expression, suggesting functional relevance. Genes that are expressed in high copy numbers by many cells encode proteins with a more general cell biological role in addition to antimicrobial and/or immunomodulatory functions.

### Investigation of the Expression of Genes Encoding Proteins of Immune Cell Processes

**Figure 3:**
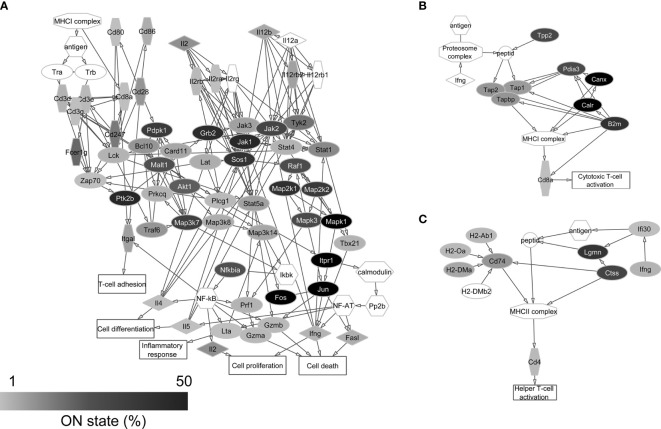
Protein-protein interaction networks of CD8+ T cell activation **(A)**, MHC class I **(B)**, and MHC class II **(C)** antigen presentation. Gray scale shows the percentage of neurons that expresses the transcript of each protein in the single-cell sequencing data (ON state frequency). The transcripts of proteins with white label were not found in the sequenced neurons. It can be seen that mainly signaling protein genes of primary importance are expressed in the majority of neurons.

**Figure 4:**
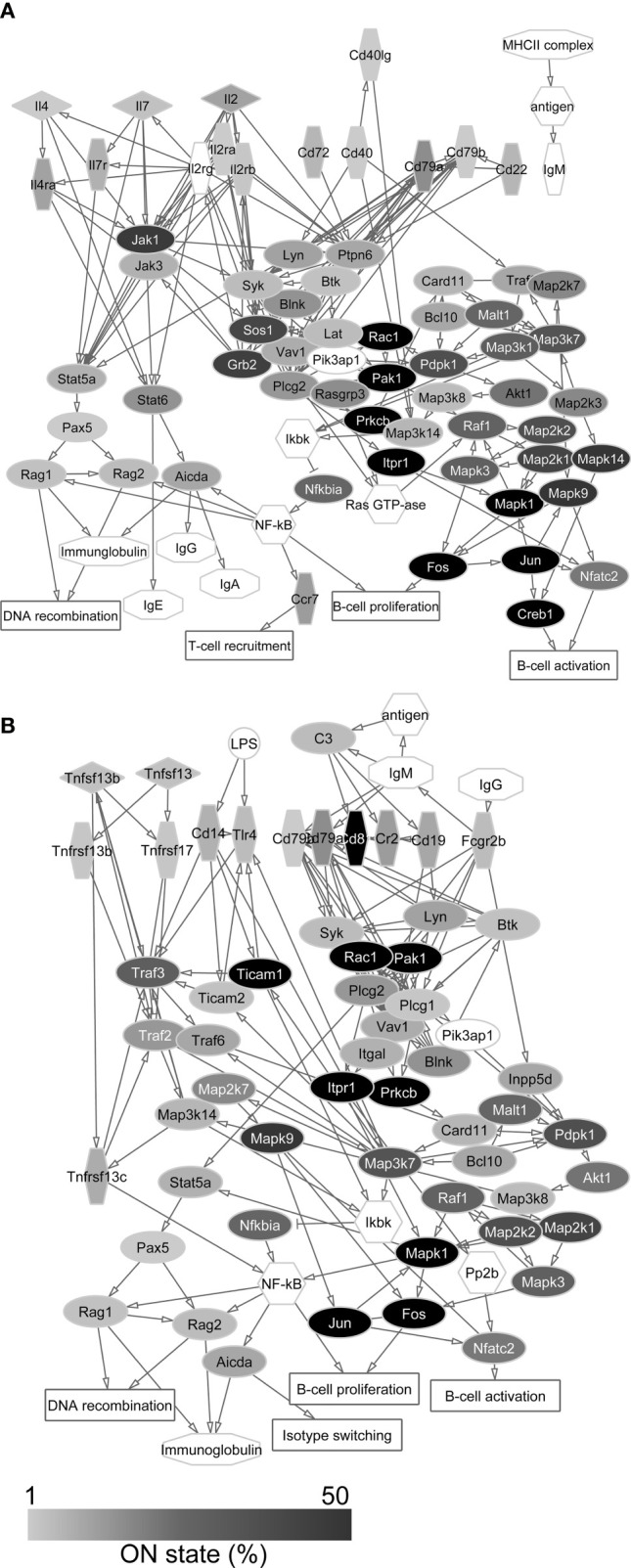
Protein-protein interaction networks of T cell dependent **(A)** and T cell independent **(B)** B cell activation. Gray scale shows the percentage of neurons that expresses the transcript of each protein in the single-cell sequencing data (ON state frequency). The transcripts of proteins with white label were not found in the sequenced neurons. In these networks, genes encoding signal regulatory proteins show high ON state frequencies as well.

Based on the reference list of immunological processes (accessible in the Curated Pathways of Pathway Studio), we identified several mRNAs in the transcriptome of PFC neurons that encode proteins playing role in immune cell communication and activation mechanisms (**Supplementary Table 2**). On **Figures 3**, **4** we show the functional protein networks and label the ON state frequency of the corresponding genes in our dataset by color scale.

**Figure 5:**
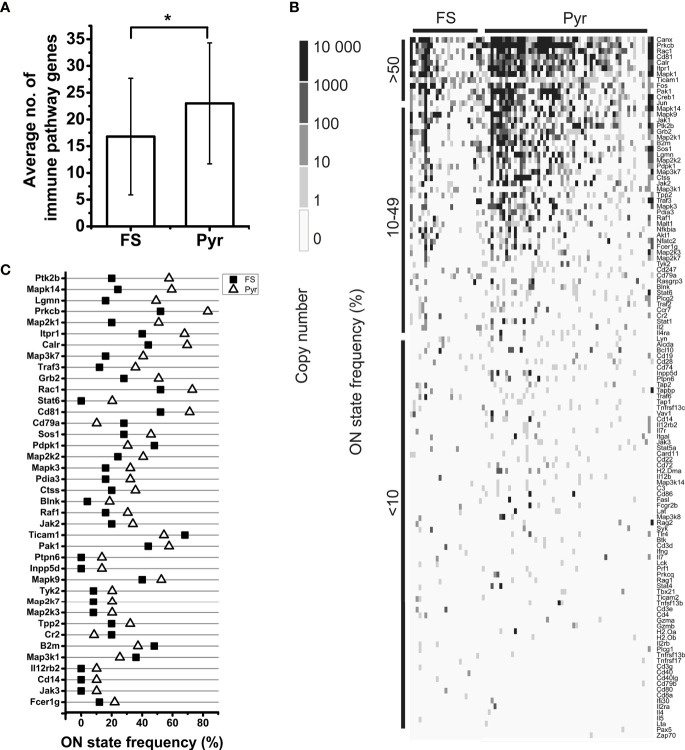
Analysis of mRNA expression of immune process proteins by FS and Pyr cells. Bar plot shows the average number of expressed immune pathway genes in FS and Pyr cells, error bars represent SD; Pyr cells expressed significantly higher number of immune pathway genes (*P* = 0.022, two-sample *t*-test) **(A)**. Heatmap of immune pathway gene expression in single neurons; genes are ordered based on their ON state frequencies; gray scale shows the copy number of mRNAs in each cell **(B)**. ON state frequencies of genes in FS and Pyr cells are shown; only those genes are listed, which have at least 10% difference in their ON state frequencies between the two cell types **(C)**. *P < 0.05.

In the case of T cell dependent B cell activation, we found the transcripts of 96.8% of the proteins in the sequencing data. For the protein networks of T cell independent B cell activation and CD8+ T cell activation, this ratio was 98.2% and 91.9%, respectively. In the case of antigen presentation pathways, similarly high coverage (100% for MHC class I and 90.0% for MHC class II antigen presentation) was found in the neuronal transcriptomes (**Supplementary Table 2**). The individual neurons expressed averagely 21.1±11.5 different genes encoding proteins of immune cell processes, showing significant differences between Pyr (23.0±11.3) and FS (16.8±10.9) cells (*P* = 0.022, two-sample *t*-test) (**Figure 5A**).

The ON state frequency of the identified 122 (calculated without overlaps between networks) immune process genes was somewhat higher than in the case of AMP genes (**Figure 5B**). We identified 9.8% of the transcripts in at least 50% of the neurons. These transcripts encode proteins mostly with basic cell biological functions (e.g., *Jun*, *Fos*, *Calr*, *Canx*, *Mapk1*, and *Creb1*); however, some of the corresponding proteins also play a significant immunological role (e.g., *Cd81* and *Ticam1*). Nearly one-third (33.6%) of the immune process genes were transcribed in 10-49% of the neurons encoding proteins with miscellaneous functions. Some of them (e.g., *Plcg2*, *Akt1*, *Pdpk1*, *Raf1*, *Mapk3*, *Map3k7*, and *Grb2*) can be associated with general cellular processes, while others (e.g., *B2m*, *Nfatc2*, *Ccr7*, *Cd79a*, *Blnk*, and *Traf3*) have well-documented immunological functions as well. The majority of immune process genes (56.6% of them) were expressed by less than 10% of the 84 neurons. Several of them (e.g., *H2-Ob*, *H2-Oa*, *H2-Dma*, *Cd4*, *Cd8a*, and *Ccl4*) encode proteins with specific immunological functions (for further details see **Supplementary Table 2**). The average ON state frequency of genes encoding proteins of immune cell processes is somewhat higher in Pyr (17.8±20.8%) than in FS (13.0±16.4%) cells. Significant (*P* = 1.2*10^-17^) positive correlation was found also in the case of these transcripts between the ON state frequencies and average normalized copy numbers (Spearman correlation coefficient: *r_S_* = 0.68, **Supplementary Figure 2**). The average normalized copy numbers of immune process transcripts were found to range from 0.1 to 1375.2 (averagely 118.2, **Supplementary Table 2** contains the data of each gene).

**Table 2** shows the cell type distribution of transcripts encoding proteins of immune cell processes. Several (48.4%) of them were evenly expressed in both types of neurons. These genes mainly encode proteins with general signaling roles in mammalian cells (e.g., components of the MAP kinase pathway, *Jak2*, *Ptk2b*, *Plcg1*, and *Itpr1*), but there are also a number of immunologically important genes among them (e.g., *Cd19*, *Cd247*, *Cd28*, *Il7*, *Ticam1*, and *Tnfrsf17*). We found that 21.3% of the immune process genes showed cell type-enriched expression. These genes also encode proteins with miscellaneous (general and/or immunological) functions (e.g., *Rac1* and *Blnk* enriched in Pyr cells or *Akt1, Cr2*, and *Cd3d* enriched in FS cells). The remaining nearly 30% of the investigated genes were transcribed in a cell type-specific manner. These include many genes encoding proteins with well-known immunological functions (e.g., *H2-Dma*, *H2-Oa*, *Il12rb2, Cd14*, and *Cd86* expressed only in some Pyr cells and *Cd3e*, *Il2rb*, and *Il5* expressed only in some FS cells). Also, the ON state frequencies of *Blnk* (4.0% of FS and 18.6% of Pyr cells) and *Cr2* (20.0% of FS and 8.5% of Pyr cells) differed between cell types. In addition, *Il12rb2*, and *Cd14* were not detected in FS cells, but were expressed in 10.2% of Pyr cells (**Figure 5C**). We found three times more immune process genes expressed only in Pyr cells, than genes expressed only in FS cells; however, it should be noted that the size of a Pyr cell is about twice as large as an FS cell.

**Table 2:** The cell type distribution of transcripts encoding proteins of immune cell processes.

Genes expressed in Pyr and FS cells			Genes expressed only in Pyr or FS cells	
Less than tenfold differences in copy numbers	More than tenfold higher copy numbers in Pyr cells	More than tenfold higher copy numbers in FS cells	Expressed only in Pyr cells	Expressed only in FS cells
*Aicda*, *B2m*, *Bcl10*, *Calr*, *Canx*, *Card11*, *Cd19*, *Cd247*, *Cd28*, *Cd74*, *Cd79a*, *Cd81*, *Creb1*, *Ctss*, *Fcer1g*, *Grb2*, *Ifng*, *Il2*, *Il4ra*, *Il7*, *Il7r*, *Itpr1*, *Jak1*, *Jak2*, *Lck*, *Lgmn*, *Lyn*, *Malt1*, *Map2k1*, *Map2k2*, *Map2k3*, *Map2k7*, *Map3k1*, *Map3k14*, *Map3k7*, *Mapk1*, *Mapk3*, *Mapk9*, *Nfatc2*, *Pak1*, *Pdia3*, *Pdpk1*, *Plcg1*, *Plcg2*, *Prkcb*, *Ptk2b*, *Raf1*, *Rag1*, *Rasgrp3*, *Tap1*, *Ticam1*, *Ticam2*, *Tnfrsf13b*, *Tnfrsf13c*, *Tnfrsf17*, *Traf2*, *Traf3*, *Tyk2*, *Vav1*	*Blnk*, *Ccr7*, *Fcgr2b*, *Itgal*, *Jun*, *Lat*, *Map3k8*, *Mapk14*, *Rac1*, *Rag2*, *Sos1*, *Stat1*, *Stat4*, *Tapbp*, *Tpp2*	*Akt1*, *Cd3d*, *Cr2*, *Fasl*, *Fos*, *Il12b*, *Nfkbia*, *Stat5a*, *Syk*, *Tap2*, *Traf6*	*Btk*, *C3*, *Cd14*, *Cd22*, *Cd72*, *Cd79b*, *Cd86*, *Gzma*, *Gzmb*, *H2.Dma*, *H2.Oa*, *H2.Ob*, *Ifi30*, *Il12rb2*, *Il2ra*, *Il4*, *Inpp5d*, *Jak3*, *Lta*, *Pax5*, *Prf1*, *Prkcq*, *Ptpn6*, *Stat6*, *Tbx21*, *Tlr4*, *Tnfsf13b*, *Zap70*	*Cd3e*, *Cd3g*, *Cd4*, *Cd40*, *Cd40lg*, *Cd80*, *Cd8a*, *Il2rb*, *Il5*
**48.4%**	**12.3%**	**9.0%**	**23.0%**	**7.4%**

The majority of genes were transcribed in both types of neurons, and several of them showed cell type-enriched expression. Around one-third of the genes coding immune cell signaling proteins were expressed in a cell type-specific manner. We compared the average copy numbers calculated on the basis of ON state cells (i.e., non-zero values), as described in the Materials and Methods.

Bold values indicate the percentage of genes belonging to the group.

Thus, the network analysis of transcripts encoding proteins of immune cell communication and activation pathways supported the high coverage of these processes in neurons at mRNA level. The average coverage of the five pathways was about 95% in the entire database of the 84 neurons and 18% in single cells, i.e., the majority of genes were transcribed only in fractions of cells. We also revealed that genes encoding hub proteins, which are the keys for functioning of the protein networks, were expressed in several neurons; however, these proteins also have more general functions, not merely immunological relevance.

### Comparison of Our Results to Previously Published Transcriptomic Data

We reviewed how many of the AMP and immune protein transcripts identified in prefrontal neurons are included in the ISH and single-cell sequencing datasets of Allen Brain Map (see **Supplementary Tables 1**, **2** for each gene). Thirty-eight percent of AMP transcripts were found in the ISH data from different brain regions. These transcripts have higher than average ON state frequencies (22.1% compared to 10.8%) and copy numbers (171.0 compared to 75.3) in our sequencing data. Of the transcripts encoding proteins of immune cell processes, 45.1% were found in the ISH data, also with ON state frequencies and copy numbers higher than the overall average (25.7% compared to 16.4% and 185.1 compared to 118.2, respectively). The single-cell sequencing database of Allen Brain Map contains the transcripts we identified with greater overlap: 46.0% of the AMP and 72.1% of the immune protein encoding genes were expressed in at least one of the neurons included in the database.

We compared the AMP and immune process gene expression levels in immune cells and hippocampal neurons on the basis of available single-cell databases to our single-neuron sequencing data and found that the ON state frequencies of the investigated genes do not differ extraordinarily (**Supplementary Table 3**, **4**). The average ON state frequency of AMP genes ranged from 3.8 ± 12.5% to 16.9 ± 27.8% in different immune cell datasets, compared to 11.1 ± 16.9% in our data. In the case of immune process genes, the average ON state frequencies ranged from 6.6 ± 13.3% to 38.7 ± 32.6% in the reviewed immune cell datasets and we found 16.4 ± 19.0% in our data. Interestingly, the mouse hippocampal neurons expressed the investigated genes to a noticeable greater extent than the prefrontal neurons we examined (17.2 ± 26.5% average ON state frequency for AMP genes and 26.1 ± 28.5% for immune process genes). This observation draws attention to the brain region-specific character of neuronal gene expression and further suggests the functional significance of the expression of immunological genes by CNS neurons. Thus, based on these comparative data, transcriptomic similarities can be found between neurons and immune cells regarding the expression of AMP and immune process genes; however, functional conclusions can only be drawn to a limited extent due to the uncertainties in the relationship between cellular mRNA and protein levels (48).

## Discussion

### The Use of Patch-Seq in the Study of Molecular Processes of CNS Neurons

We report here on mRNAs of AMPs with immunomodulatory function and proteins involved in lymphocyte activation and antigen presentation mechanisms expressed by prefrontal Pyr and FS cells harvested after electrophysiological identification. Some of the described mRNAs has not been detected in cortical neurons before, probably due to the lower sensitivity of immunostaining and ISH, as well as low-depth sequencing of large number of cells compared to ultra-deep sequencing. We emphasize that the linear amplification of cDNA combined with ultra-deep sequencing up to more than 20 million reads is an extremely sensitive method to detect transcripts with low copy numbers in single-cell samples. It is in correspondence with the fact that we were able to recover more than 19,000 transcripts from less than 90 neurons.

Because of the high labor demand and costs of the method, the number of cells sequenced was low but not remarkably lower than in other similar patch-seq studies (e.g., 46, 49), allowing to apply the highest quality sequencing technology. We performed a normalization procedure described earlier (30) to make single-cell sequencing data more reliable and ensure the significant positive correlation between total normalized read number and reconstructed transcripts. We also reviewed available datasets, as the ISH and single-cell databases of Allen Brain Map (24, 25) and single-cell data of immune cells and hippocampal neurons (for GEO accession numbers and references see Materials and Methods) to compare the expression pattern of AMP and immune process genes in neurons and immune cells obtained by different transcriptomic technologies. The genes included in the ISH data of Allen Brain Map showed remarkably higher ON state frequencies and normalized copy numbers than the overall average, suggesting the accuracy of our sequencing and data analysis protocols. Due to the high sensitivity of the applied mRNA amplification and sequencing technology, we uncovered many AMP and immune process transcripts, which were not detected in the single-cell transcriptomics data of Allen Brain Map. On the other hand (probably as a consequence of the methodological approach somewhat similar to ours), several of the immunological transcripts we identified in PFC neurons, could also be detected in the murine hippocampus. Based on these results, it can be hypothesized, that the neuronal expression of AMP and immune cell process genes may indeed play an important role in nervous system processes (e.g., in direct neuro-immune interactions). The revealed transcriptomic similarities (see **Supplementary Tables 3**, **4**) of the selected genes in CNS neurons and immune cells also support this idea. However, considering the notable differences in experimental (e.g., different species, cell numbers and sequencing techniques) and data analysis methods, the results of qualitative and/or quantitative comparison of different transcriptomic datasets need to be interpreted critically.

### Functional Interpretation of Transcriptomic Data

As it can be seen in **Figures 2B**, **5B**, as well as in **Supplementary Tables 1**, **2**, many transcripts could be identified only in small fractions of cells with low average copy numbers. However, comparing the expression data of genes encoding neuronal proteins mentioned as examples in the Results section to the data of AMP and immune process genes, it can be seen that there are no substantial differences that definitely rule out the functional relevance of the neuronal expression of immune system genes. Moreover, it is also known that in biological samples the majority of genes are weakly expressed, and only a fraction of them are expressed in high copy numbers (50). Owing to the bursting kinetics of gene expression (51), at a certain time point several genes can be in OFF state and some others in ON state (52). The transcriptional regulation (e.g., the frequency and the duration of ON and OFF states) shows gene-specific features (53, 54) and there is not yet enough knowledge about general regulatory mechanisms to reliably estimate the cellular protein levels from mRNA copy numbers. Ultimately, protein abundances correlate positively with the mRNA copy numbers to some extent (55, 56) in a gene and cell type-specific manner (57) and the degree of correlation may depend also on the actual condition of the cells (58). Thus, in general it can be established that increase in the copy number of a particular mRNA initiates an increase in the level of the encoded protein (54). Nonetheless, it should also be emphasized, that the presence of a transcript in a cell does not automatically imply that the corresponding protein can be found in a functional form as well (e.g., due to translational and posttranslational regulatory mechanisms). Detection of the encoded proteins would be relevant for functional analysis of immune system-related transcripts; however, single-cell proteomics can detect only proteins of highest abundances, which is usually not more than 1,000-1,200 different proteins in a cell (27).

Accordingly, transcript copy number differences between Pyr and FS cells can be interpreted so that potentially the protein levels also differ to a certain degree; moreover, the cell type-specific or enriched expression of many of the investigated genes may suggests that the detection of immune transcripts in neurons could not derive from sequencing artifacts or negligible stochastic gene expression noise. The functional analysis of differential expression of immune genes in principal cells and interneurons of the mammalian neocortex is a promising field of future research.

Interestingly, the neuronal ON state frequencies of the investigated genes are comparable to the patterns observed in immune cells (**Supplementary Tables 3**, **4**), proposing that the transcriptional regulation of these genes may be based on similar mechanisms in the two cell types. Since the reviewed databases contain heterogeneous data sets, with different experimental and data processing approaches, the assumption about similarities between neurons and immune cells needs verification directly at the protein level.

### The Potential Roles of Immune Gene Expression in CNS Neurons

Several AMPs also exhibiting well documented immunomodulatory activities (59, 60) were expressed in a cell type-specific or enriched manner, especially in Pyr cells (**Table 1**). It suggests an important role for Pyr cells in the control and modulation of immune processes in the CNS. The expression of some AMP genes (e.g., *Camp*, *Defb9*, *Defb11*, and *Defb35*) in the brain was documented in earlier studies at the mRNA (61) and even at the protein level (62); however, these results do not include information on the exact cellular sources of AMPs. Interestingly, it was reported in *Drosophila melanogaster* that the neuron-specific inhibition of the expression of GNBP-like3 (a peptide with antibacterial activity) resulted in the impairment of long-term memory (63), suggesting that AMPs expressed by neurons may participate in nervous system-specific processes. On the other hand, it is well established, that several neuropeptides (e.g., proenkephalin A, neurokinin-1, neuropeptide Y, and even amyloid-β) having primarily neurobiological functions, also possess antimicrobial activity and are often termed as neuro-antimicrobial peptides (NAMPs) (64, 65). The antimicrobial activity of amyloid-β has supported the hypothesis that chronic CNS infections may contribute to the early onset of Alzheimer’s disease *via* the overproduction of AMPs such as amyloid-β itself (66). In summary, the neuronal expression of AMP genes presented here draws attention to the possibility that, in addition to AMPs produced mainly by endothelial and glial cells, the same peptides expressed by cortical neurons may also contribute to the diverse CNS functions of these host defense molecules (antimicrobial, immunomodulatory and signaling activities, as reviewed by 65).

The network analysis of immune system-related proteins encoded by their identified transcripts in prefrontal neurons showed that components of T and B cell activation and antigen presentation processes can be recovered in the single-neuron sequencing data to a notable extent. The observed high coverage of immune cell pathways in the neuronal transcriptomes raises the possibility of functional crosstalk between neurons and CNS infiltrating T and B cells (67, 68). Thus, our data reveal the putative basics of molecular neuro-immune interactions and suggest initiating lines of investigations to uncover the details of direct interplay between neurons and immune cells. The proteins encoded by the identified transcripts could have multiple functions as well, so it cannot be excluded that the immune cell-related molecules and processes play different roles in neurons than in lymphocytes or glial cells. The neuronal expression of several proteins of primary immunological relevance (e.g., MHC class I molecules, complement components) and their roles in physiological and pathological CNS processes have been described (69–71). There are also data suggesting that glial cells and neurons can present antigens to T cells entering the brain parenchyma under certain conditions (72, 73). The presence of components of antigen presentation processes in the transcriptome of PFC neurons also supports this idea.

In conclusion, we were able to detect the mRNAs of AMPs, as well as of proteins of T and B cell activation and antigen presentation processes in prefrontal Pyr and FS cells by ultra-deep sequencing of single-cell samples. The similarities in gene expression pattern between neurons and immune cells along with the remarkable coverage of immune cell-specific molecular pathways in the neuronal transcriptome suggest putting more effort on the investigation of direct interactions between neurons and lymphocytes and contribution of neurons to immunomodulation in the brain by AMPs. Furthermore, we recommend the reanalysis of CNS omics data focusing on immune system-related mRNAs and proteins. Our results presented here provide a potential molecular substrate for the direct contacts between neurons and T cells observed earlier (18, 23). We emphasize that our study is only a first step toward understanding the functional importance of direct neuro-immune interactions in the brain and we propose initiating further research on that field.

## Data Availability Statement

The original contributions presented in the study are included in the article/**Supplementary Material**. Further inquiries can be directed to the corresponding author.

## Author Contributions

DM, JK, JG, and KK conceptualized the study. LR performed the experiments. DM, VT, AM, JK, JG, and KK analyzed the data. DM, VT, AM, LR, JK, JG, and KK wrote the manuscript, and all co-authors reviewed the paper and agreed to the published version of the manuscript.

## Funding

This study was supported by the National Research, Development and Innovation Office of Hungary (grants KTIA_NAP_13-2-2014-0017 and 2017-1.2.1-NKP-2017-00002, FIEK_16-1-2016-0005) to DM, VT, AM, LR, JK, GJ, and KK, János Bolyai research scholarship to AM, grant PD135510 to AM; 2017-2.3.7-TÉT-IN-2017-00038 Innovative drug development against dementia to LR and GJ; research scholarship of Gedeon Richter Plc. Centenarial Foundation, Budapest, Hungary to DM.

## Conflict of Interest

Authors LR and GJ were employed by CRU Hungary Ltd. Authors GJ and KAK were employed by InnoScience Ltd.

The remaining authors declare that the research was conducted in the absence of any commercial or financial relationships that could be construed as a potential conflict of interest.

## Publisher’s Note

All claims expressed in this article are solely those of the authors and do not necessarily represent those of their affiliated organizations, or those of the publisher, the editors and the reviewers. Any product that may be evaluated in this article, or claim that may be made by its manufacturer, is not guaranteed or endorsed by the publisher.

## References

[1] Lampron A ElAli A Rivest S. Innate Immunity in the CNS: Redefining the Relationship Between the CNS and Its Environment. Neuron (2013) 78:214–32. doi: 10.1016/j.neuron.2013.04.005 23622060

[2] Filiano AJ Gadani SP Kipnis J. How and Why do T Cells and Their Derived Cytokines Affect the Injured and Healthy Brain? Nat Rev Neurosci (2017) 18:375–84. doi: 10.1038/nrn.2017.39 PMC5823005 28446786

[3] Miłkowska P Popko K Demkow U Wolańczyk T. Pro-Inflammatory Cytokines in Psychiatric Disorders in Children and Adolescents: A Review. Adv Exp Med Biol (2017) 1021:73–80. doi: 10.1007/5584_2017_32 28405892

[4] Lee C-H Giuliani F. The Role of Inflammation in Depression and Fatigue. Front Immunol (2019) 10:1696. doi: 10.3389/fimmu.2019.01696 31379879 PMC6658985

[5] Williams WM Torres S Siedlak SL Castellani RJ Perry G Smith MA. Antimicrobial Peptide β-Defensin-1 Expression Is Upregulated in Alzheimer’s Brain. J Neuroinflamm (2013) 10:1–11. doi: 10.1186/1742-2094-10-127 PMC3817866 24139179

[6] Yang D Chertov O Bykovskaia SN Chen Q Buffo MJ Shogan J. B-Defensins: Linking Innate and Adaptive Immunity Through Dendritic and T Cell CCR6. Science (1999) 286:525–8. doi: 10.1126/science.286.5439.525 10521347

[7] Yang D Chen Q Chertov O Oppenheim JJ. Human Neutrophil Defensins Selectively Chemoattract Naive T and Immature Dendritic Cells. J Leukoc Biol (2000) 68:9–14. doi: 10.1189/jlb.68.1.9 10914484

[8] Hao HN Zhao J Lotoczky G Grever WE Lyman WD. Induction of Human Beta-Defensin-2 Expression in Human Astrocytes by Lipopolysaccharide and Cytokines. J Neurochem (2001) 77:1027–35. doi: 10.1046/j.1471-4159.2001.00305.x 11359868

[9] Sawada M Itoh Y Suzumura A Marunouchi T. Expression of Cytokine Receptors in Cultured Neuronal and Glial Cells. Neurosci Lett (1993) 160:131–4. doi: 10.1016/0304-940(93)90396-3 8247342

[10] Ericsson A Liu C Hart RP Sawchenko PE. Type 1 Interleukin-1 Receptor in the Rat Brain: Distribution, Regulation, and Relationship to Sites of IL-1-Induced Cellular Activation. J Comp Neurol (1995) 361:681–98. doi: 10.1002/cne.903610410 8576422

[11] Figiel I. Pro-Inflammatory Cytokine TNF-Alpha as a Neuroprotective Agent in the Brain. Acta Neurobiol Exp (Wars) (2008) 68:526–34. 10.55782/ane-2008-1720 19112477

[12] Hodo TW Prudente de Aquino MT Shimamoto A Shanker A. Critical Neurotransmitters in the Neuroimmune Network. Front Immunol (2020) 11:1869. doi: 10.3389/fimmu.2020.01869 32973771 PMC7472989

[13] Francelin C Veneziani LP dos Santos Farias A Mendes-da-Cruz DA Savino W. Neurotransmitters Modulate Intrathymic T Cell Development. Front Cell Dev Biol (2021) 9:668067. doi: 10.3389/fcell.2021.668067 33928093 PMC8076891

[14] Anthony IC Crawford DH Bell JE. B Lymphocytes in the Normal Brain: Contrasts With HIV-Associated Lymphoid Infiltrates and Lymphomas. Brain (2003) 126:1058–67. doi: 10.1093/brain/awg118 12690046

[15] Yshii L Gebauer C Bernard-Valnet R Liblau R. Neurons and T Cells: Understanding This Interaction for Inflammatory Neurological Diseases. Eur J Immunol (2015) 45:2712–20. doi: 10.1002/eji.201545759 26345279

[16] Ortega SB Torres VO Latchney SE Whoolery CW Noorbhai IZ Poinsatte K. B Cells Migrate Into Remote Brain Areas and Support Neurogenesis and Functional Recovery After Focal Stroke in Mice. Proc Natl Acad Sci USA (2020) 117:4983–93. doi: 10.1073/pnas.1913292117 PMC7060723 32051245

[17] Meuth SG Herrmann AM Simon OJ Siffrin V Melzer N Bittner S. Cytotoxic CD8+ T Cell–Neuron Interactions: Perforin-Dependent Electrical Silencing Precedes But Is Not Causally Linked to Neuronal Cell Death. J Neurosci (2009) 29:15397–409. doi: 10.1523/JNEUROSCI.4339-09.2009 PMC6666122 20007464

[18] Liblau RS Gonzalez-Dunia D Wiendl H Zipp F. Neurons as Targets for T Cells in the Nervous System. Trends Neurosci (2013) 36:315–24. doi: 10.1016/j.tins.2013.01.008 23478065

[19] Daglas M Draxler DF Ho H McCutcheon F Galle A Au AE. Activated CD8 ^+^ T Cells Cause Long-Term Neurological Impairment After Traumatic Brain Injury in Mice. Cell Rep (2019) 29:1178–91. doi: 10.1016/j.celrep.2019.09.046 31665632

[20] Meng H Zhao H Cao X Hao J Zhang H Liu Y. Double-Negative T Cells Remarkably Promote Neuroinflammation After Ischemic Stroke. Proc Natl Acad Sci USA (2019) 116:5558–63. doi: 10.1073/pnas.1814394116 PMC6431175 30819895

[21] Méndez-Samperio P. Recent Advances in the Field of Antimicrobial Peptides in Inflammatory Diseases. Adv Biomed Res (2013) 2:50. doi: 10.4103/2277-9175.114192 24516850 PMC3905337

[22] Williams WM Castellani RJ Weinberg A Perry G Smith MA. Do β-Defensins and Other Antimicrobial Peptides Play a Role in Neuroimmune Function and Neurodegeneration? ScientificWorldJournal (2012) 2012:905785. doi: 10.1100/2012/905785 22606066 PMC3346844

[23] Nitsch R Pohl EE Smorodchenko A Infante-Duarte C Aktas O Zipp F. Direct Impact of T Cells on Neurons Revealed by Two-Photon Microscopy in Living Brain Tissue. J Neurosci (2004) 24:2458–64. doi: 10.1523/JNEUROSCI.4703-03.2004 PMC6729479 15014121

[24] Allen Institute for Brain Science. Allen Mouse Brain Atlas (2004). Available at: https://mouse.brain-map.org/.

[25] Allen Institute for Brain Science. Allen Cell Types Database (2015). Available at: https://portal.brain-map.org/atlases-and-data/rnaseq.

[26] Ponomarenko EA Poverennaya EV Ilgisonis EV Pyatnitskiy MA Kopylov AT Zgoda VG. The Size of the Human Proteome: The Width and Depth. Int J Anal Chem (2016) 2016:7436849. doi: 10.1155/2016/7436849 27298622 PMC4889822

[27] Kelly RT. Single-Cell Proteomics: Progress and Prospects, Mol. Cell Proteomics (2020) 19:1739–48. doi: 10.1074/mcp.R120.002234 PMC7664119 32847821

[28] Morris J Singh JM Eberwine JH. Transcriptome Analysis of Single Cells. J Vis Exp (2011) 50:2634. doi: 10.3791/2634 PMC3376915 21540826

[29] Malboeuf CM Yang X Charlebois P Qu J Berlin AM Casali M. Complete Viral RNA Genome Sequencing of Ultra-Low Copy Samples by Sequence-Independent Amplification. Nucleic Acids Res (2013) 41:e13. doi: 10.1093/nar/gks794 22962364 PMC3592391

[30] Ravasz L Kékesi KA Mittli D Todorov MI Borhegyi Z Ercsey-Ravasz M. Cell Surface Protein mRNAs Show Differential Transcription in Pyramidal and Fast-Spiking Cells as Revealed by Single-Cell Sequencing. Cereb Cortex (2021) 31:731–45. doi: 10.1093/cercor/bhaa195 PMC7906784 32710103

[31] Van Gelder RN von Zastrow ME Yool A Dement WC Barchas JD Eberwine JH. Amplified RNA Synthesized From Limited Quantities of Heterogeneous cDNA. Proc Natl Acad Sci USA (1990) 87:1663–7. doi: 10.1073/pnas.87.5.1663 PMC53542 1689846

[32] Eberwine J Yeh H Miyashiro K Cao Y Nair S Finnell R. Analysis of Gene Expression in Single Live Neurons. Proc Natl Acad Sci USA (1992) 89:3010–4. doi: 10.1073/pnas.89.7.3010 PMC48793 1557406

[33] Chubb JR Liverpool TB. Bursts and Pulses: Insights From Single Cell Studies Into Transcriptional Mechanisms. Curr Opin Genet Dev (2010) 20:478–84. doi: 10.1016/j.gde.2010.06.009 20638837

[34] Suter DM Molina N Gatfield D Schneider K Schibler U Naef F. Mammalian Genes Are Transcribed With Widely Different Bursting Kinetics. Science (2011) 332:472–4. doi: 10.1126/science.1198817 21415320

[35] Kumar A Doan VM Kunkli B Csősz É. Construction of Unified Human Antimicrobial and Immunomodulatory Peptide Database and Examination of Antimicrobial and Immunomodulatory Peptides in Alzheimer’s Disease Using Network Analysis of Proteomics Datasets. Front Genet (2021) 12:633050. doi: 10.3389/fgene.2021.633050 33995478 PMC8113759

[36] Nikitin A Egorov S Daraselia N Mazo I. Pathway Studio–the Analysis and Navigation of Molecular Networks. Bioinformatics (2003) 19:2155–7. doi: 10.1093/bioinformatics/btg290 14594725

[37] Szklarczyk D Gable AL Lyon D Junge A Wyder S Huerta-Cepas J. STRING V11: Protein-Protein Association Networks With Increased Coverage, Supporting Functional Discovery in Genome-Wide Experimental Datasets. Nucleic Acid Res (2019) 47:D607–13. doi: 10.1093/nar/gky1131 PMC6323986 30476243

[38] Shannon P Markiel A Ozier O Baliga NS Wang JT Ramage D. Cytoscape: A Software Environment for Integrated Models of Biomolecular Interaction Networks. Genome Res (2003) 13:2498–504. doi: 10.1101/gr.1239303 PMC403769 14597658

[39] Nirschl CJ Suárez-Fariñas M Izar B Prakadan S Dannenfelser R Tirosh I. Ifnγ-Dependent Tissue-Immune Homeostasis Is Co-Opted in the Tumor Microenvironment. Cell (2017) 170:127–41. doi: 10.1016/j.cell.2017.06.016 PMC5569303 28666115

[40] Zhang L Yu X Zheng L Zhang Y Li Y Fang Q. Lineage Tracking Reveals Dynamic Relationships of T Cells in Colorectal Cancer. Nature (2018) 564:268–72. doi: 10.1038/s41586-018-0694-x 30479382

[41] Povoleri GA Nova-Lamperti E Scottà C Fanelli G Chen YC Becker PD. Human Retinoic Acid–Regulated CD161+ Regulatory T Cells Support Wound Repair in Intestinal Mucosa. Nat Immunol (2018) 19:1403–14. doi: 10.1038/s41590-018-0230-z PMC6474659 30397350

[42] Sade-Feldman M Yizhak K Bjorgaard SL Ray JP de Boer CG Jenkins RW. Defining T Cell States Associated With Response to Checkpoint Immunotherapy in Melanoma. Cell (2018) 175:998–1013. doi: 10.1016/j.cell.2018.10.038 30388456 PMC6641984

[43] Snyder ME Finlayson MO Connors TJ Dogra P Senda T Bush E. Generation and Persistence of Human Tissue-Resident Memory T Cells in Lung Transplantation. Sci Immunol (2019) 4:1–16. doi: 10.1126/sciimmunol.aav5581 PMC6435356 30850393

[44] Szabo PA Levitin HM Miron M Snyder ME Senda T Yuan J. Single-Cell Transcriptomics of Human T Cells Reveals Tissue and Activation Signatures in Health and Disease. Nat Commun (2019) 10:1–16. doi: 10.1038/s41467-019-12464-3 31624246 PMC6797728

[45] Zheng C Zheng L Yoo JK Guo H Zhang Y Guo X. Landscape of Infiltrating T Cells in Liver Cancer Revealed by Single-Cell Sequencing. Cell (2017) 169:1342–56. doi: 10.1016/j.cell.2017.05.035 28622514

[46] Földy C Darmanis S Aoto J Malenka RC Quake SR Südhof TC. Single-Cell RNAseq Reveals Cell Adhesion Molecule Profiles in Electrophysiologically Defined Neurons. Proc Natl Acad Sci USA (2016) 113:E5222–31. doi: 10.1073/pnas.1610155113 PMC5024636 27531958

[47] Munsky B Neuert G Van Oudenaarden A. Using Gene Expression Noise to Understand Gene Regulation. Science (2012) 336:183–7. doi: 10.1126/science.1216379 PMC3358231 22499939

[48] Lin J Amir A. Homeostasis of Protein and mRNA Concentrations in Growing Cells. Nat Commun (2018) 9:4496. doi: 10.1038/s41467-018-06714-z 30374016 PMC6206055

[49] Fuzik J Zeisel A Máté Z Calvigioni D Yanagawa Y Szabó G. Integration of Electrophysiological Recordings With Single-Cell RNA-Seq Data Identifies Neuronal Subtypes. Nat Biotechnol (2016) 34:175–83. doi: 10.1038/nbt.3443 PMC4745137 26689544

[50] Chen Y Kamat V Dougherty ER Bittner ML Meltzer PS Trent JM. Ratio Statistics of Gene Expression Levels and Applications to Microarray Data Analysis. Bioinformatics (2002) 18:1207–15. doi: 10.1093/bioinformatics/18.9.1207 12217912

[51] Tunnacliffe E Chubb JR. What Is a Transcriptional Burst? Trends Genet (2020) 36:288–97. doi: 10.1016/j.tig.2020.01.003 32035656

[52] Li C Cesbron F Oehler M Brunner M Höfer T. Frequency Modulation of Transcriptional Bursting Enables Sensitive and Rapid Gene Regulation. Cell Syst (2018) 6:409–23. doi: 10.1016/j.cels.2018.01.012 29454937

[53] Emerson BM. Specificity of Gene Regulation. Cell (2002) 109:267–70. doi: 10.1016/s0092-8674(02)00740-7 12015975

[54] Liu Y Beyer A Aebersold R. On the Dependency of Cellular Protein Levels on mRNA Abundance. Cell (2016) 165:535–50. doi: 10.1016/j.cell.2016.03.014 27104977

[55] Guo Y Xiao P Lei S Deng F Xiao GG Liu Y. How Is mRNA Expression Predictive for Protein Expression? A Correlation Study on Human Circulating Monocytes. Acta Biochim Biophys Sin (Sanghai) (2008) 40:426–36. doi: 10.1111/j.1745-7270.2008.00418.x 18465028

[56] Maier T Güell M Serrano L. Correlation of mRNA and Protein in Complex Biological Samples. FEBS Lett (2009) 583:3966–73. doi: 10.1016/j.febslet.2009.10.036 19850042

[57] Pascal LE True LD Campbell DS Deutsch EW Risk M Coleman IM. Correlation of mRNA and Protein Levels: Cell Type-Specific Gene Expression of Cluster Designation Antigens in the Prostate. BMC Genomics (2008) 9:1–13. doi: 10.1186/1471-2164-9-246 18501003 PMC2413246

[58] Koussounadis A Langdon SP Um IH Harrison DJ Smith VA. Relationship Between Differentially Expressed mRNA and mRNA-Protein Correlations in a Xenograft Model System. Sci Rep (2015) 5:1–9. doi: 10.1038/srep10775 PMC4459080 26053859

[59] Bowdish DME Davidson DJ Scott MG Hancock REW. Immunomodulatory Activities of Small Host Defense Peptides. Antimicrob Agents Chemother (2005) 49:1727–32. doi: 10.1128/AAC.49.5.1727-1732.2005 PMC1087655 15855488

[60] Hilchie AL Wuerth K Hancock RE. Immune Modulation by Multifaceted Cationic Host Defense (Antimicrobial) Peptides. Nat Chem Biol (2013) 9:761–8. doi: 10.1038/nchembio.1393 24231617

[61] Maxwell AI Morrison GM Dorin JR. Rapid Sequence Divergence in Mammalian β-Defensins by Adaptive Evolution. Mol Immunol (2003) 40:413–21. doi: 10.1016/s0161-5890(03)00160-3 14568387

[62] Bergman P Termén S Johansson L Nyström L Arenas E Jonsson AB. The Antimicrobial Peptide rCRAMP Is Present in the Central Nervous System of the Rat. J Neurochem (2005) 93:1132–40. doi: 10.1111/j.1471-4159.2005.03081.x 15934934

[63] Barajas-Azpeleta R Wu J Gill J Welte R Seidel C McKinney S. Antimicrobial Peptides Modulate Long-Term Memory. PloS Genet (2018) 14:e1007440. doi: 10.1371/journal.pgen.1007440 30312294 PMC6224176

[64] Schluesener HJ Su Y Ebrahimi A Pouladsaz D. Antimicrobial Peptides in the Brain: Neuropeptides and Amyloid. Front Biosci (2012) 4:1375–80. doi: 10.2741/s339 22652879

[65] Su Y Zhang K Schluesener HJ. Antimicrobial Peptides in the Brain. Arch Immunol Ther Exp (2010) 58:365–77. doi: 10.1007/s00005-010-0089-7 20668978

[66] Welling MM Nabuurs RJ van der Weerd L. Potential Role of Antimicrobial Peptides in the Early Onset of Alzheimer’s Disease. Alzheimers Dement (2015) 11:51–7. doi: 10.1016/j.jalz.2013.12.020 24637300

[67] Smolders J Heutinck KM Fransen NL Remmerswaal EBM Hombrink P Ten Berge IJM. Tissue-Resident Memory T Cells Populate the Human Brain. Nat Commun (2018) 9:4593. doi: 10.1038/s41467-018-07053-9 30389931 PMC6214977

[68] Tanabe S Yamashita T. B-1a Lymphocytes Promote Oligodendrogenesis During Brain Development. Nat Neurosci (2018) 21:506–16. doi: 10.1038/s41593-018-0106-4 29507409

[69] Boulanger LM. Immune Proteins in Brain Development and Synaptic Plasticity. Neuron (2009) 64:93–109. doi: 10.1016/j.neuron.2009.09.001 19840552

[70] Nelson PA Sage JR Wood SC Davenport CM Anagnostaras SG Boulanger LM. MHC Class I Immune Proteins Are Critical for Hippocampus-Dependent Memory and Gate NMDAR-Dependent Hippocampal Long-Term Depression. Learn Mem (2013) 20:505–17. doi: 10.1101/lm.031351.113 PMC3744042 23959708

[71] Hammad A Westacott L Zaben M. The Role of the Complement System in Traumatic Brain Injury: A Review. J Neuroinflamm (2018) 15:1–15. doi: 10.1186/s12974-018-1097-5 PMC5778697 29357880

[72] Cebrián C Loike JD Sulzer D. Neuronal MHC-I Expression and its Implications in Synaptic Function, Axonal Regeneration and Parkinson’s and Other Brain Diseases. Front Neuroanat (2014) 8:114. doi: 10.3389/fnana.2014.00114 25352786 PMC4195363

[73] Schetters ST Gomez-Nicola D Garcia-Vallejo JJ Van Kooyk Y. Neuroinflammation: Microglia and T Cells Get Ready to Tango. Front Immunol (2018) 8:1905. doi: 10.3389/fimmu.2017.01905 29422891 PMC5788906

