# Supplementary material for: The Single-Cell Transcriptomic Analysis of Prefrontal Pyramidal Cells and Interneurons Reveals the Neuronal Expression of Genes Encoding Antimicrobial Peptides and Immune Proteins
Source: Front Immunol. 2021 Oct 25;12:749433. doi: 10.3389/fimmu.2021.749433 (PMC8574171; doi:10.3389/fimmu.2021.749433)
Supplement: Supplementary file 1 [file DataSheet_1.docx]

**Supplementary Material**

**Supplementary Figures and Tables**





**Supplementary Figure 1.** The figure shows the average normalized copy number of the identified AMP transcripts per cell as a function of their ON state frequency. As it can be seen, higher ON state frequencies are mostly associated with higher copy numbers, resulting in a significant (P = 2.6*10^-13^) positive correlation (Spearman correlation coefficient: r_S_ = 0.65).





**Supplementary Figure 2.** The figure shows the average normalized copy number of transcripts encoding immune process proteins as a function of their ON state frequency. As it can be seen, higher ON state frequencies are mostly associated with higher copy numbers, resulting in a significant (P = 1.2*10^-17^) positive correlation (Spearman correlation coefficient: r_S_ = 0.68).

**Supplementary Table 1.** The AMP transcripts identified in the transcriptomes of prefrontal pyramidal cells and interneurons based on the reference list of Kumar et al. (2021). The table shows the ON state frequency and average copy number of each gene, as well as whether it can be found in the in situ hybridization (ISH) and single-cell sequencing datasets of Allen Brain Map (Allen Mouse Brain Atlas, 2004; Allen Cell Types Database, 2015).

| **Antimicrobial and immunomodulatory protein (AMP) genes** | | **ON state (%)** | **Average copy number per cell** | **Allen Brain Map ISH data** | **Allen Brain Map single-cell data** |
| --- | --- | --- | --- | --- | --- |
| Adm | adrenomedullin | 1.2 | 1.4 | No | Yes |
| Ang2 | angiogenin-2 | 2.4 | 0.2 | No | No |
| Ang4 | angiogenin-4 | 2.4 | 0.1 | No | No |
| Apobec3 | DNA dC → dU-editing enzyme APOBEC-3G | 7.1 | 1.9 | No | Yes |
| App | amyloid-beta precursor protein | 83.3 | 942.3 | Yes | Yes |
| Atp5f1 | ATP synthase subunit beta, mitochondrial | 51.2 | 320.0 | Yes | Yes |
| B2m | beta-2-microglobulin | 40.5 | 285.4 | Yes | Yes |
| Bpifa1 | BPI fold-containing family A member 1 precursor | 2.4 | 0.7 | No | No |
| Bpifa2 | BPI fold-containing family A member 2 | 1.2 | 0.5 | No | No |
| Bpifb4 | BPI fold-containing family B member 4 | 2.4 | 1.0 | No | Yes |
| Bst2 | bone marrow stromal antigen 2 | 1.2 | 0.1 | No | Yes |
| Calca | calcitonin gene-related peptide 1 | 3.6 | 0.5 | No | Yes |
| Camp | cathelicidin antimicrobial peptide | 2.4 | 0.3 | No | No |
| Ccl4 | C-C motif chemokine 4 | 8.3 | 383.4 | No | Yes |
| Ccl17 | C-C motif chemokine 17 | 2.4 | 13.7 | No | Yes |
| Ccl22 | C-C motif chemokine 22 | 2.4 | 1.0 | No | No |
| Ccl24 | C-C motif chemokine 24 | 2.4 | 1.0 | No | No |
| Ccl25 | C-C motif chemokine 25 | 6.0 | 0.6 | No | Yes |
| Ccl27a | C-C motif chemokine 27 | 50.0 | 315.5 | Yes | Yes |
| Ccl28 | C-C motif chemokine 28 | 3.6 | 0.2 | No | Yes |
| Chga | chromogranin-A | 33.3 | 128.1 | Yes | Yes |
| Clu | clusterin | 46.4 | 92.8 | Yes | Yes |
| Cxcl1 | growth-regulated alpha protein | 3.6 | 164.2 | No | No |
| Cxcl2 | C-X-C motif chemokine 2 | 7.1 | 82.8 | Yes | No |
| Cxcl3 | C-X-C motif chemokine 3 | 1.2 | 2.7 | No | No |
| Cxcl9 | C-X-C motif chemokine 9 | 1.2 | 0.1 | No | No |
| Cxcl10 | C-X-C motif chemokine 10 | 3.6 | 36.3 | No | No |
| Cxcl11 | C-X-C motif chemokine 11 | 3.6 | 0.1 | No | No |
| Cxcl12 | stromal cell-derived factor 1 | 23.8 | 133.4 | Yes | Yes |
| Cxcl13 | C-X-C motif chemokine 13 | 1.2 | 0.2 | No | No |
| Cxcl14 | C-X-C motif chemokine 14 | 9.5 | 19.5 | Yes | Yes |
| Defb1 | beta-defensin 1 | 3.6 | 0.5 | No | Yes |
| Defb2 | beta-defensin 2 | 1.2 | 2.0 | No | No |
| Defb3 | beta-defensin 3 | 1.2 | 0.2 | No | No |
| Defb5 | beta-defensin 5 | 1.2 | 1.4 | No | No |
| Defb11 | beta-defensin 11 | 1.2 | 0.1 | No | No |
| Defb13 | beta-defensin 13 | 1.2 | 0.1 | No | No |
| Defb15 | beta-defensin 15 | 1.2 | 80.0 | No | No |
| Defb28 | beta-defensin 28 | 1.2 | 0.4 | No | No |
| Defb29 | beta-defensin 29 | 1.2 | 0.9 | No | No |
| Defb30 | beta-defensin 30 | 3.6 | 2.2 | No | No |
| Defb35 | beta-defensin 35 | 3.6 | 2.3 | No | No |
| Dlc1 | Rho GTPase-activating protein 7 | 44.0 | 132.3 | Yes | Yes |
| Dmbt1 | deleted in malignant brain tumors 1 protein | 8.3 | 0.3 | No | No |
| F2 | prothrombin | 1.2 | 2.0 | Yes | No |
| Fau | 40S ribosomal protein S30 | 61.9 | 245.7 | Yes | Yes |
| Fga | fibrinogen alpha chain | 3.6 | 0.4 | Yes | Yes |
| Flg2 | filaggrin-2 | 1.2 | 0.2 | No | No |
| Furin | furin | 28.6 | 2.1 | Yes | Yes |
| Galp | galanin-like peptide | 1.2 | 0.3 | No | Yes |
| Gapdh | glyceraldehyde-3-phosphate dehydrogenase | 59.5 | 39.0 | Yes | Yes |
| Gbp1 | guanylate-binding protein 1 | 3.6 | 0.5 | No | No |
| Gbp2 | guanylate-binding protein 2 | 1.2 | 1.2 | No | No |
| Gpr15 | protein GPR15L | 1.2 | 0.9 | No | No |
| H2afj | histone H2A.J | 35.7 | 136.9 | No | Yes |
| Hist1h2bc | histone H2B type 1-C/E/F/G/I | 20.2 | 24.6 | Yes | Yes |
| Hmgn2 | non-histone chromosomal protein HMG-17 | 33.3 | 14.2 | Yes | Yes |
| Hp | haptoglobin beta chain | 1.2 | 0.3 | Yes | No |
| Hrnr | hornerin | 4.8 | 0.5 | No | No |
| Ifih1 | interferon-induced helicase C domain-containing protein 1 | 15.5 | 0.9 | No | Yes |
| Ifnl3 | interferon lambda-3 | 1.2 | 1.8 | No | No |
| Iqgap2 | Ras GTPase-activating-like protein IQGAP2 | 21.4 | 168.8 | Yes | Yes |
| Isg20 | interferon-stimulated gene 20 kDa protein | 8.3 | 5.9 | No | No |
| Klk5 | kallikrein-5 | 1.2 | 0.3 | No | No |
| Kng1 | kininogen-1 | 4.8 | 0.4 | Yes | Yes |
| Leap2 | liver-expressed antimicrobial peptide 2 | 1.2 | 4.4 | No | No |
| Lgals3 | galectin-3 | 11.9 | 1.2 | No | Yes |
| Ltf | lactotransferrin | 1.2 | 0.2 | No | Yes |
| Lyz1 | lysozyme C-1 | 1.2 | 0.8 | Yes | No |
| Lyz2 | lysozyme C-2 | 9.5 | 3.5 | Yes | Yes |
| Mx1 | interferon-induced GTP-binding protein Mx1 | 7.1 | 2.9 | No | No |
| Npy | neuropeptide Y | 9.5 | 1063.9 | Yes | Yes |
| Nts | neurotensin | 1.2 | 1.4 | Yes | Yes |
|  |  |  |  |  |  |
| Pglyrp3 | peptidoglycan recognition protein 3 | 3.6 | 0.6 | No | Yes |
| Pglyrp4 | peptidoglycan recognition protein 4 | 3.6 | 0.5 | No | No |
| Pigr | polymeric immunoglobulin receptor | 14.3 | 1.4 | Yes | No |
| Pla2g2a | phospholipase A2, membrane associated | 1.2 | 1.0 | Yes | No |
| Pomc | melanocyte-stimulating hormone alpha (Pro-opiomelanocortin) | 3.6 | 193.9 | Yes | Yes |
| Ppbp | platelet Basic protein | 3.6 | 1.1 | No | No |
| Prf1 | perforin-1 | 3.6 | 0.3 | No | No |
| Prg2 | bone marrow proteoglycan | 1.2 | 4.1 | No | No |
| Rarres2 | retinoic acid receptor responder protein 2 | 3.6 | 6.1 | Yes | No |
| Retn | resistin | 3.6 | 0.4 | Yes | Yes |
| Rnase6 | ribonuclease K6 | 1.2 | 0.3 | Yes | No |
| Romo1 | reactive oxygen species modulator 1 | 36.9 | 560.6 | Yes | Yes |
| S100a7a | protein S100-A7A | 1.2 | 0.3 | No | No |
| S100a9 | protein S100-A9 | 1.2 | 0.2 | Yes | No |
| Serpina1c | alpha-1-antitrypsin 1-3 | 1.2 | 0.2 | No | No |
| Serpina1e | alpha-1-antitrypsin 1-5 | 1.2 | 4.2 | No | No |
| Serpind1 | heparin cofactor 2 | 2.4 | 12.4 | No | Yes |
| Snca | alpha-synuclein | 48.8 | 410.9 | Yes | Yes |
| Sp1 | transcription factor Sp1 | 21.4 | 142.6 | No | Yes |
| Spag11b | sperm-associated antigen 11B | 1.2 | 0.5 | No | No |
| Tac1 | protachykinin-1 | 11.9 | 1012.4 | Yes | Yes |
| Tor2a | prosalusin | 11.9 | 51.6 | Yes | Yes |
| Vgf | neurosecretory protein VGF | 45.2 | 236.1 | Yes | Yes |
| Vip | VIP peptides | 9.5 | 5.5 | Yes | Yes |
| Wfdc12 | WAP four-disulfide core domain protein12 | 7.1 | 1.1 | No | No |
| Xcl1 | lymphotactin | 1.2 | 0.4 | Yes | No |
| Zg16 | zymogen granule protein 16 homolog B | 1.2 | 1.4 | No | No |

**Supplementary Table 2.** The transcripts encoding proteins of immune cell processes detected in the cytoplasm of prefrontal pyramidal cells and interneurons. The table contains the genes of all proteins involved in the reviewed immune cell communication and activation processes based on the Curated Pathways library of Elsevier Pathway Studio v11.0 (Nikitin et al., 2003). ON state frequencies and average copy numbers of each gene are shown, as well as whether the gene can be found in the in situ hybridization (ISH) and single-cell transcriptomic datasets of Allen Brain Map (Allen Mouse Brain Atlas, 2004; Allen Cell Types Database, 2015).

| **MHC class I antigen presentation** | | **ON state (%)** | **Average copy number per cell** | **Allen Brain Map ISH data** | **Allen Brain Map single-cell data** |
| --- | --- | --- | --- | --- | --- |
| Tap2 | transporter 2, ATP-binding cassette, sub-family B (MDR/TAP) | 9.5 | 3.6 | No | Yes |
| Ifng | interferon, gamma | 3.6 | 0.3 | No | No |
| Canx | calnexin | 77.4 | 385.9 | Yes | Yes |
| Calr | calreticulin | 61.9 | 138.2 | Yes | Yes |
| Cd8a | CD8a molecule | 1.2 | 0.4 | Yes | No |
| Pdia3 | protein disulfide isomerase family A, member 3 | 27.4 | 25.5 | Yes | Yes |
| Tapbp | TAP binding protein (tapasin) | 9.5 | 19.0 | Yes | Yes |
| B2m | beta-2-microglobulin | 40.5 | 285.4 | Yes | Yes |
| Tap1 | transporter 1, ATP-binding cassette, sub-family B (MDR/TAP) | 8.3 | 0.3 | No | No |
| Tpp2 | tripeptidyl peptidase II | 28.6 | 81.3 | No | Yes |
| **MHC class II antigen presentation** | | | | | |
| Ifng | interferon, gamma | 3.6 | 0.3 | No | No |
| H2-Ob | major histocompatibility complex, class II, DO beta | 2.4 | 0.7 | No | No |
| H2-Oa | major histocompatibility complex, class II, DO alpha | 2.4 | 67.8 | No | No |
| H2-DMb2 | major histocompatibility complex, class II, DM beta | 0.0 | 0.0 | Not searched | Not searched |
| H2-Dma | major histocompatibility complex, class II, DM alpha | 6.0 | 11.6 | No | Yes |
| Cd4 | CD4 molecule | 2.4 | 1.0 | Yes | Yes |
| Lgmn | legumain | 39.3 | 270.4 | Yes | Yes |
| Cd74 | CD74 molecule, major histocompatibility complex, class II invariant chain | 9.5 | 0.3 | No | Yes |
| Ctss | cathepsin S | 31.0 | 1162.1 | Yes | Yes |
| Ifi30 | interferon, gamma-inducible protein 30 | 1.2 | 6.0 | No | Yes |
|  |  |  |  |  |  |
|  |  |  |  |  |  |
|  |  |  |  |  |  |
| **T cell dependent B cell activation** | | **ON state (%)** | **Average copy number per cell** | **Allen Brain Map ISH data** | **Allen Brain Map single-cell data** |
| Plcg2 | phospholipase C, gamma 2 (phosphatidylinositol-specific) | 13.1 | 0.8 | No | Yes |
| Pdpk1 | 3-phosphoinositide dependent protein kinase 1 | 35.7 | 31.5 | Yes | Yes |
| Map3k8 | mitogen-activated protein kinase kinase kinase 8 | 4.8 | 124.6 | No | Yes |
| Traf6 | TNF receptor-associated factor 6, E3 ubiquitin protein ligase | 9.5 | 27.8 | Yes | Yes |
| Prkcb | protein kinase C, beta | 73.8 | 1375.2 | Yes | Yes |
| Nfatc2 | nuclear factor of activated T-cells, cytoplasmic 2 | 20.2 | 33.8 | Yes | Yes |
| Map2k3 | mitogen-activated protein kinase kinase 3 | 16.7 | 123.4 | No | Yes |
| Lyn | LYN proto-oncogene, Src family tyrosine kinase | 10.7 | 9.5 | Yes | Yes |
| Pak1 | p21 protein (Cdc42/Rac)-activated kinase 1 | 53.6 | 928.2 | Yes | Yes |
| Mapk1 | mitogen-activated protein kinase 1 | 58.3 | 368.7 | Yes | Yes |
| Map3k14 | mitogen-activated protein kinase kinase kinase 14 | 6.0 | 0.3 | No | Yes |
| Map2k7 | mitogen-activated protein kinase kinase 7 | 16.7 | 8.1 | Yes | Yes |
| Cd72 | CD72 molecule | 6.0 | 1.4 | Yes | Yes |
| Creb1 | cAMP responsive element binding protein 1 | 52.4 | 212.3 | No | Yes |
| Grb2 | growth factor receptor-bound protein 2 | 44.0 | 57.2 | Yes | Yes |
| Bcl10 | B-cell CLL/lymphoma 10 | 9.5 | 66.3 | No | Yes |
| Itpr1 | inositol 1,4,5-trisphosphate receptor, type 1 | 59.5 | 550.2 | Yes | Yes |
| Il2rg | interleukin 2 receptor, gamma | 0.0 | 0.0 | Not searched | Not searched |
| Ccr7 | chemokine (C-C motif) receptor 7 | 11.9 | 7.9 | No | No |
| Akt1 | v-akt murine thymoma viral oncogene homolog 1 | 21.4 | 122.6 | Yes | Yes |
| Stat6 | signal transducer and activator of transcription 6, interleukin-4 induced | 14.3 | 14.1 | Yes | Yes |
| Cd22 | CD22 molecule | 6.0 | 0.2 | No | No |
| Aicda | activation-induced cytidine deaminase | 9.5 | 0.8 | No | No |
| Mapk14 | mitogen-activated protein kinase 14 | 48.8 | 723.2 | No | Yes |
| Il4ra | interleukin 4 receptor | 10.7 | 43.1 | No | Yes |
|  |  |  |  |  |  |
|  |  |  |  |  |  |
| Pik3ap1 | phosphoinositide-3-kinase adaptor protein 1 | 0.0 | 0.0 | Not searched | Not searched |
| Il7r | interleukin 7 receptor | 7.1 | 0.9 | No | No |
| Jak3 | Janus kinase 3 | 7.1 | 0.5 | No | Yes |
| Map3k1 | mitogen-activated protein kinase kinase kinase 1 | 28.6 | 2.4 | Yes | Yes |
| Il7 | interleukin 7 | 3.6 | 8.1 | No | Yes |
| Pax5 | paired box 5 | 1.2 | 0.8 | No | No |
| Rag1 | recombination activating gene 1 | 3.6 | 0.5 | No | No |
| Stat5a | signal transducer and activator of transcription 5A | 7.1 | 0.8 | No | Yes |
| Vav1 | vav 1 guanine nucleotide exchange factor | 8.3 | 26.1 | No | No |
| Map2k2 | mitogen-activated protein kinase kinase 2 | 35.7 | 108.9 | No | Yes |
| Mapk9 | mitogen-activated protein kinase 9 | 48.8 | 327.7 | Yes | Yes |
| Map2k1 | mitogen-activated protein kinase kinase 1 | 41.7 | 743.4 | Yes | Yes |
| Il2rb | interleukin 2 receptor, beta | 2.4 | 0.3 | Yes | No |
| Rag2 | recombination activating gene 2 | 4.8 | 42.3 | No | No |
| Lat | linker for activation of T cells | 4.8 | 240.2 | No | No |
| Cd40lg | CD40 ligand | 1.2 | 0.5 | No | No |
| Sos1 | son of sevenless homolog 1 (Drosophila) | 40.5 | 449.5 | Yes | Yes |
| Cd40 | CD40 molecule, TNF receptor superfamily member 5 | 1.2 | 2.0 | Yes | Yes |
| Il2ra | interleukin 2 receptor, alpha | 1.2 | 38.6 | No | No |
| Cd79b | CD79b molecule, immunoglobulin-associated beta | 1.2 | 0.1 | No | No |
| Jak1 | Janus kinase 1 | 46.4 | 96.7 | No | Yes |
| Nfkbia | nuclear factor of kappa light polypeptide gene enhancer in B-cells inhibitor, alpha | 25.0 | 264.6 | Yes | Yes |
| Card11 | caspase recruitment domain family, member 11 | 6.0 | 0.3 | No | Yes |
| Rac1 | ras-related C3 botulinum toxin substrate 1 | 66.7 | 553.0 | Yes | Yes |
| Raf1 | Raf-1 proto-oncogene, serine/threonine kinase | 26.2 | 99.8 | Yes | Yes |
| Cd79a | CD79a molecule, immunoglobulin-associated alpha | 15.5 | 1.8 | Yes | No |
| Btk | Bruton agammaglobulinemia tyrosine kinase | 3.6 | 0.3 | Yes | Yes |
| Blnk | B-cell linker | 14.3 | 9.1 | Yes | Yes |
|  |  |  |  |  |  |
| Rasgrp3 | RAS guanyl releasing protein 3 (calcium and DAG-regulated) | 15.5 | 240.3 | No | Yes |
| Mapk3 | mitogen-activated protein kinase 3 | 27.4 | 51.5 | Yes | Yes |
| Il4 | interleukin 4 | 1.2 | 0.1 | No | Yes |
| Map3k7 | mitogen-activated protein kinase kinase kinase 7 | 33.3 | 110.4 | Yes | Yes |
| Ptpn6 | protein tyrosine phosphatase, non-receptor type 6 | 9.5 | 155.2 | Yes | Yes |
| Il2 | interleukin 2 | 10.7 | 10.5 | No | No |
| Syk | spleen tyrosine kinase | 4.8 | 11.5 | No | Yes |
| Jun | jun proto-oncogene | 51.2 | 175.3 | Yes | Yes |
| Fos | FBJ murine osteosarcoma viral oncogene homolog | 53.6 | 802.6 | Yes | Yes |
| Malt1 | MALT1 paracaspase | 25.0 | 24.1 | Yes | Yes |
| **T cell independent B cell activation** | | **ON state (%)** | **Average copy number per cell** | **Allen Brain Map ISH data** | **Allen Brain Map single-cell data** |
| Plcg2 | phospholipase C, gamma 2 (phosphatidylinositol-specific) | 13.1 | 0.8 | No | Yes |
| Cd81 | CD81 molecule | 65.5 | 239.9 | Yes | Yes |
| Plcg1 | phospholipase C, gamma 1 | 2.4 | 0.8 | Yes | Yes |
| Ticam2 | toll-like receptor adaptor molecule 2 | 3.6 | 0.9 | Yes | Yes |
| Tnfrsf17 | tumor necrosis factor receptor superfamily, member 17 | 2.4 | 0.2 | No | No |
| Pdpk1 | 3-phosphoinositide dependent protein kinase 1 | 35.7 | 31.5 | Yes | Yes |
| Map3k8 | mitogen-activated protein kinase kinase kinase 8 | 4.8 | 124.6 | No | Yes |
| Traf6 | TNF receptor-associated factor 6, E3 ubiquitin protein ligase | 9.5 | 27.8 | Yes | Yes |
| Prkcb | protein kinase C, beta | 73.8 | 1375.2 | Yes | Yes |
| Nfatc2 | nuclear factor of activated T-cells, cytoplasmic, calcineurin-dependent 2 | 20.2 | 33.8 | Yes | Yes |
| C3 | complement component 3 | 4.8 | 0.4 | No | No |
| Lyn | LYN proto-oncogene, Src family tyrosine kinase | 10.7 | 9.5 | Yes | Yes |
| Pak1 | p21 protein (Cdc42/Rac)-activated kinase 1 | 53.6 | 928.2 | Yes | Yes |
| Tlr4 | toll-like receptor 4 | 4.8 | 17.8 | No | No |
| Mapk1 | mitogen-activated protein kinase 1 | 58.3 | 368.7 | Yes | Yes |
| Map3k14 | mitogen-activated protein kinase kinase kinase 14 | 6.0 | 0.3 | No | Yes |
| Map2k7 | mitogen-activated protein kinase kinase 7 | 16.7 | 8.1 | Yes | Yes |
| Bcl10 | B-cell CLL/lymphoma 10 | 9.5 | 66.3 | No | Yes |
| Itpr1 | inositol 1,4,5-trisphosphate receptor, type 1 | 59.5 | 550.2 | Yes | Yes |
| Akt1 | v-akt murine thymoma viral oncogene homolog 1 | 21.4 | 122.6 | Yes | Yes |
| Tnfsf13b | tumor necrosis factor (ligand) superfamily, member 13b | 3.6 | 631.3 | No | Yes |
| Aicda | activation-induced cytidine deaminase | 9.5 | 0.8 | No | No |
| Traf2 | TNF receptor-associated factor 2 | 13.1 | 1.6 | Yes | Yes |
| Pik3ap1 | phosphoinositide-3-kinase adaptor protein 1 | 0.0 | 0.0 | Not searched | Not searched |
| Traf3 | TNF receptor-associated factor 3 | 28.6 | 176.8 | No | Yes |
| Pax5 | paired box 5 | 1.2 | 0.8 | No | No |
| Rag1 | recombination activating gene 1 | 3.6 | 0.5 | No | No |
| Stat5a | signal transducer and activator of transcription 5A | 7.1 | 0.8 | No | Yes |
| Vav1 | vav 1 guanine nucleotide exchange factor | 8.3 | 26.1 | No | No |
| Map2k2 | mitogen-activated protein kinase kinase 2 | 35.7 | 108.9 | No | Yes |
| Mapk9 | mitogen-activated protein kinase 9 | 48.8 | 327.7 | Yes | Yes |
| Map2k1 | mitogen-activated protein kinase kinase 1 | 41.7 | 743.4 | Yes | Yes |
| Rag2 | recombination activating gene 2 | 4.8 | 42.3 | No | No |
| Tnfrsf13b | tumor necrosis factor receptor superfamily, member 13B | 2.4 | 0.3 | Yes | Yes |
| Cd79b | CD79b molecule, immunoglobulin-associated beta | 1.2 | 0.1 | No | No |
| Nfkbia | nuclear factor of kappa light polypeptide gene enhancer in B-cells inhibitor, alpha | 25.0 | 264.6 | Yes | Yes |
| Card11 | caspase recruitment domain family, member 11 | 6.0 | 0.3 | No | Yes |
| Ticam1 | toll-like receptor adaptor molecule 1 | 58.3 | 4.2 | No | Yes |
| Rac1 | ras-related C3 botulinum toxin substrate 1 | 66.7 | 553.0 | Yes | Yes |
| Raf1 | Raf-1 proto-oncogene, serine/threonine kinase | 26.2 | 99.8 | Yes | Yes |
| Cd79a | CD79a molecule, immunoglobulin-associated alpha | 15.5 | 1.8 | Yes | No |
| Cd14 | CD14 molecule | 7.1 | 88.5 | No | No |
| Btk | Bruton agammaglobulinemia tyrosine kinase | 3.6 | 0.3 | Yes | Yes |
| Blnk | B-cell linker | 14.3 | 9.1 | Yes | Yes |
| Mapk3 | mitogen-activated protein kinase 3 | 27.4 | 51.5 | Yes | Yes |
| Map3k7 | mitogen-activated protein kinase kinase kinase 7 | 33.3 | 110.4 | Yes | Yes |
| Cd19 | CD19 molecule | 9.5 | 0.6 | No | No |
| Fcgr2b | Fc fragment of IgG, low affinity IIb, receptor (CD32) | 4.8 | 53.5 | No | No |
| Syk | spleen tyrosine kinase | 4.8 | 11.5 | No | Yes |
| Tnfsf13b | tumor necrosis factor (ligand) superfamily, member 13B | 3.6 | 631.3 | No | Yes |
| Inpp5d | inositol polyphosphate-5-phosphatase, 145kDa | 9.5 | 72.6 | No | Yes |
| Cr2 | complement component (3d/Epstein Barr virus) receptor 2 | 11.9 | 9.4 | No | Yes |
| Tnfrsf13c | tumor necrosis factor receptor superfamily, member 13C | 8.3 | 0.4 | No | Yes |
| Jun | jun proto-oncogene | 51.2 | 175.3 | Yes | Yes |
| Fos | FBJ murine osteosarcoma viral oncogene homolog | 53.6 | 802.6 | Yes | Yes |
| Malt1 | MALT1 paracaspase | 25.0 | 24.1 | Yes | Yes |
| **CD8+ T cell activation** | | **ON state (%)** | **Average copy number per cell** | **Allen Brain Map ISH data** | **Allen Brain Map single-cell data** |
| Cd86 | CD86 molecule | 4.8 | 240.3 | No | No |
| Ifng | interferon, gamma | 3.6 | 0.3 | No | No |
| Tcra | T cell receptor alpha locus | 0.0 | 0.0 | Not searched | Not searched |
| Plcg1 | phospholipase C, gamma 1 | 2.4 | 0.8 | Yes | Yes |
| Tcrb | T cell receptor beta locus | 0.0 | 0.0 | Not searched | Not searched |
| Pdpk1 | 3-phosphoinositide dependent protein kinase 1 | 35.7 | 31.5 | Yes | Yes |
| Map3k8 | mitogen-activated protein kinase kinase kinase 8 | 4.8 | 124.6 | No | Yes |
| Traf6 | TNF receptor-associated factor 6, E3 ubiquitin protein ligase | 9.5 | 27.8 | Yes | Yes |
| Tbx21 | T-box 21 | 3.6 | 0.7 | No | Yes |
| Mapk1 | mitogen-activated protein kinase 1 | 58.3 | 368.7 | Yes | Yes |
| Cd3e | CD3e molecule, epsilon (CD3-TCR complex) | 2.4 | 35.8 | No | No |
| Map3k14 | mitogen-activated protein kinase kinase kinase 14 | 6.0 | 0.3 | No | Yes |
| Grb2 | growth factor receptor-bound protein 2 | 44.0 | 57.2 | Yes | Yes |
| Itgal | integrin, alpha L (antigen CD11A (p180), lymphocyte function-associated antigen 1 | 7.1 | 18.7 | No | No |
| Il5 | interleukin 5 | 1.2 | 0.3 | No | Yes |
| Bcl10 | B-cell CLL/lymphoma 10 | 9.5 | 66.3 | No | Yes |
| Itpr1 | inositol 1,4,5-trisphosphate receptor, type 1 | 59.5 | 550.2 | Yes | Yes |
| Gzma | granzyme A (granzyme 1, cytotoxic T-lymphocyte-associated serine esterase 3) | 2.4 | 1.1 | No | No |
| Il2rg | interleukin 2 receptor, gamma | 0.0 | 0.0 | Not searched | Not searched |
| Fcer1g | Fc fragment of IgE, high affinity I, receptor for | 19.0 | 245.4 | No | Yes |
| Cd8a | CD8a molecule | 1.2 | 0.4 | Yes | No |
| Akt1 | v-akt murine thymoma viral oncogene homolog 1 | 21.4 | 122.6 | Yes | Yes |
| Cd247 | CD247 molecule | 15.5 | 1.3 | Yes | Yes |
| Gzmb | granzyme B (granzyme 2, cytotoxic T-lymphocyte-associated serine esterase 1) | 2.4 | 0.9 | Yes | No |
| Jak3 | Janus kinase 3 | 7.1 | 0.5 | No | Yes |
| Prf1 | perforin 1 (pore forming protein) | 3.6 | 0.3 | No | No |
| Stat1 | signal transducer and activator of transcription 1, 91kDa | 11.9 | 167.0 | Yes | Yes |
| Cd28 | CD28 molecule | 9.5 | 0.9 | Yes | No |
| Il12rb2 | interleukin 12 receptor, beta 2 | 7.1 | 0.9 | No | Yes |
| Jak2 | Janus kinase 2 | 29.8 | 250.8 | No | Yes |
| Stat5a | signal transducer and activator of transcription 5A | 7.1 | 0.8 | No | Yes |
| Cd80 | CD80 molecule | 1.2 | 4.9 | No | Yes |
| Map2k2 | mitogen-activated protein kinase kinase 2 | 35.7 | 108.9 | No | Yes |
| Il12a | interleukin 12A | 0.0 | 0.0 | Not searched | Not searched |
| Map2k1 | mitogen-activated protein kinase kinase 1 | 41.7 | 743.4 | Yes | Yes |
| Il2rb | interleukin 2 receptor, beta | 2.4 | 0.3 | Yes | No |
| Lat | linker for activation of T cells | 4.8 | 240.2 | No | No |
| Sos1 | son of sevenless homolog 1 (Drosophila) | 40.5 | 449.5 | Yes | Yes |
| Cd3g | CD3g molecule, gamma (CD3-TCR complex) | 1.2 | 0.1 | Yes | No |
| Tyk2 | tyrosine kinase 2 | 16.7 | 4.4 | No | Yes |
| Il2ra | interleukin 2 receptor, alpha | 1.2 | 38.6 | No | No |
| Jak1 | Janus kinase 1 | 46.4 | 96.7 | No | Yes |
| Nfkbia | nuclear factor of kappa light polypeptide gene enhancer in B-cells inhibitor, alpha | 25.0 | 264.6 | Yes | Yes |
| Prkcq | protein kinase C, theta | 3.6 | 25.5 | Yes | Yes |
| Card11 | caspase recruitment domain family, member 11 | 6.0 | 0.3 | No | Yes |
| Il12rb1 | interleukin 12 receptor, beta 1 | 0.0 | 0.0 | Not searched | Not searched |
| Ptk2b | protein tyrosine kinase 2 beta | 46.4 | 141.4 | Yes | Yes |
| Zap70 | zeta-chain (TCR) associated protein kinase 70kDa | 1.2 | 0.7 | Yes | Yes |
| Raf1 | Raf-1 proto-oncogene, serine/threonine kinase | 26.2 | 99.8 | Yes | Yes |
| Il12b | interleukin 12B | 6.0 | 7.1 | Yes | No |
| Stat4 | signal transducer and activator of transcription 4 | 3.6 | 40.6 | No | Yes |
| Lck | LCK proto-oncogene, Src family tyrosine kinase | 3.6 | 0.3 | No | Yes |
| Mapk3 | mitogen-activated protein kinase 3 | 27.4 | 51.5 | Yes | Yes |
| Il4 | interleukin 4 | 1.2 | 0.1 | No | Yes |
| Map3k7 | mitogen-activated protein kinase kinase kinase 7 | 33.3 | 110.4 | Yes | Yes |
| Il2 | interleukin 2 | 10.7 | 10.5 | No | No |
| Fasl | Fas ligand (TNF superfamily, member 6) | 4.8 | 28.4 | No | Yes |
| Cd3d | CD3d molecule, delta (CD3-TCR complex) | 3.6 | 13.2 | No | No |
| Jun | jun proto-oncogene | 51.2 | 175.3 | Yes | Yes |
| Fos | FBJ murine osteosarcoma viral oncogene homolog | 53.6 | 802.6 | Yes | Yes |
| Malt1 | MALT1 paracaspase | 25.0 | 24.1 | Yes | Yes |
| Lta | lymphotoxin alpha | 1.2 | 1.0 | Yes | No |

**Supplementary Table 3.** The ON state frequencies (%) of AMP genes in our single-neuron data and in online datasets obtained from immune cells and hippocampal neurons (for references see Materials and Methods in the main text). The genes are arranged in descending order based on our results. Only genes that can be identified in both species are presented. Averages are shown in the last line. (Abbreviations: PFC, prefrontal cortex; CRC, colorectal cancer; UMI, unique molecular identifier; BAL, bronchoalveolar lavage; LC, liver cancer; HPC, hippocampus.)

| **Dataset** | **Own data** | **GSE**  **100337** | **GSE**  **108989** | **GSE**  **119373** | **GSE**  **120575** | **GSE**  **124675** | **GSE**  **126030** | **GSE**  **98638** | **GSE**  **75386** |
| --- | --- | --- | --- | --- | --- | --- | --- | --- | --- |
| **Method** | **HiSeq2500/ NextSeq500** | **Smart-**  **seq2** | **Smart-seq2** | **Chromium** | **Smart-**  **seq2** | **SMARTer (UMI)** | **Chromium** | **Tang & Smart-seq2** | **NextSeq 500** |
| **Species** | **Mouse** | **Human** | **Human** | **Human** | **Human** | **Human** | **Human** | **Human** | **Mouse** |
| **Cell type** | **PFC**  **neurons** | **Mono-phago-**  **cytes** | **T cells (CRC)** | **CD4+**  **CD25+**  **T cells** | **CD45**  **cells** | **T cells (BAL)** | **T cells** | **T cells**  **(LC)** | **HPC neurons** |
| **Gene** | **ON state frequency (%)** | | | | | | | | |
| App | 83.3 | 41.1 | 4.8 | 3.2 | 7.9 | 0.6 | 5.3 | 5.0 | 89.4 |
| Fau | 61.9 | 99.7 | 98.4 | 90.4 | 90.4 | 39.3 | 99.3 | 98.8 | 84.0 |
| Gapdh | 59.5 | 99.4 | 99.7 | 70.7 | 93.2 | 16.0 | 94.2 | 99.0 | 93.6 |
| Atp5f1 | 51.2 | 0.0 | 0.0 | 0.0 | 0.0 | 0.0 | 0.0 | 0.0 | 81.9 |
| Ccl27a | 50.0 | 0.3 | 1.5 | 0.0 | 0.2 | 0.0 | 0.0 | 1.7 | 69.1 |
| Snca | 48.8 | 12.0 | 0.6 | 0.0 | 2.4 | 0.5 | 1.0 | 0.3 | 81.9 |
| Clu | 46.4 | 2.1 | 8.1 | 0.4 | 4.1 | 1.6 | 4.5 | 8.7 | 72.3 |
| Vgf | 45.2 | 0.0 | 0.1 | 0.0 | 0.1 | 0.0 | 0.1 | 0.1 | 69.1 |
| Dlc1 | 44.0 | 1.5 | 1.0 | 0.0 | 0.6 | 0.2 | 0.6 | 1.0 | 40.4 |
| B2m | 40.5 | 100.0 | 100.0 | 100.0 | 99.9 | 99.7 | 100.0 | 100.0 | 62.8 |
| Romo1 | 36.9 | 37.5 | 57.0 | 18.1 | 22.6 | 13.6 | 39.7 | 58.3 | 68.1 |
| H2afj | 35.7 | 42.3 | 28.8 | 10.3 | 16.3 | 2.0 | 8.9 | 26.3 | 61.7 |
| Chga | 33.3 | 0.3 | 0.3 | 0.0 | 0.5 | 0.0 | 0.1 | 0.5 | 67.0 |
| Hmgn2 | 33.3 | 97.9 | 89.3 | 57.3 | 72.4 | 4.3 | 45.7 | 89.6 | 72.3 |
| Furin | 28.6 | 11.1 | 27.2 | 1.7 | 12.7 | 1.9 | 11.1 | 13.2 | 26.6 |
| Cxcl12 | 23.8 | 21.3 | 0.4 | 0.0 | 0.6 | 0.0 | 0.0 | 0.7 | 10.6 |
| Iqgap2 | 21.4 | 33.3 | 58.5 | 16.6 | 30.0 | 3.4 | 11.6 | 59.8 | 50.0 |
| Sp1 | 21.4 | 29.7 | 14.0 | 3.8 | 13.8 | 2.8 | 5.3 | 19.8 | 26.6 |
| Hist1h2bc | 20.2 | 18.9 | 2.8 | 0.4 | 7.0 | 0.2 | 0.4 | 4.5 | 43.6 |
| Ifih1 | 15.5 | 25.8 | 26.6 | 2.7 | 13.2 | 1.1 | 5.7 | 26.4 | 2.1 |
| Pigr | 14.3 | 71.8 | 1.3 | 0.0 | 0.5 | 1.1 | 0.5 | 1.4 | 1.1 |
| Lgals3 | 11.9 | 63.4 | 46.1 | 16.8 | 24.6 | 15.4 | 19.5 | 26.9 | 6.4 |
| Tac1 | 11.9 | 0.0 | 0.2 | 0.0 | 0.1 | 0.0 | 0.0 | 0.2 | 27.7 |
| Tor2a | 11.9 | 31.8 | 20.8 | 3.7 | 9.1 | 1.1 | 5.8 | 21.5 | 46.8 |
| Cxcl14 | 9.5 | 0.0 | 0.2 | 0.0 | 0.1 | 40.4 | 0.0 | 0.3 | 30.9 |
| Npy | 9.5 | 0.0 | 0.1 | 0.0 | 0.0 | 0.0 | 0.0 | 0.1 | 36.2 |
| Vip | 9.5 | 0.6 | 0.2 | 0.0 | 0.0 | 0.0 | 0.0 | 0.2 | 17.0 |
| Ccl4 | 8.3 | 24.9 | 64.5 | 0.2 | 42.0 | 36.7 | 21.0 | 47.9 | 62.8 |
| Dmbt1 | 8.3 | 0.3 | 1.0 | 0.0 | 0.3 | 0.0 | 0.0 | 0.7 | 5.3 |
| Isg20 | 8.3 | 24.9 | 78.3 | 53.2 | 54.4 | 13.6 | 52.6 | 72.9 | 5.3 |
| Apobec3 | 7.1 | 30.9 | 74.2 | 11.0 | 54.4 | 20.8 | 19.9 | 74.7 | 5.3 |
| Cxcl2 | 7.1 | 18.9 | 1.0 | 0.0 | 4.3 | 0.0 | 1.5 | 0.7 | 20.2 |
| Mx1 | 7.1 | 53.5 | 44.9 | 6.3 | 28.0 | 3.5 | 17.5 | 41.9 | 0.0 |
| Wfdc12 | 7.1 | 0.3 | 0.1 | 0.0 | 0.0 | 0.0 | 0.0 | 0.1 | 1.1 |
| Ccl25 | 6.0 | 0.3 | 0.2 | 0.0 | 0.2 | 0.1 | 0.0 | 0.6 | 29.8 |
| Hrnr | 4.8 | 0.9 | 0.4 | 0.0 | 1.7 | 0.0 | 0.0 | 0.3 | 0.0 |
| Kng1 | 4.8 | 2.4 | 2.0 | 0.0 | 0.2 | 5.6 | 0.3 | 4.8 | 1.1 |
| Calca | 3.6 | 1.2 | 0.1 | 0.0 | 0.1 | 0.0 | 0.0 | 0.2 | 12.8 |
| Ccl28 | 3.6 | 26.7 | 9.8 | 4.6 | 2.8 | 1.3 | 2.4 | 9.0 | 5.3 |
| Cxcl1 | 3.6 | 8.4 | 0.6 | 0.0 | 1.0 | 0.0 | 0.6 | 0.7 | 7.4 |
| Cxcl10 | 3.6 | 10.5 | 0.6 | 0.0 | 2.2 | 0.0 | 5.4 | 1.5 | 3.2 |
| Cxcl11 | 3.6 | 4.2 | 0.3 | 0.0 | 0.8 | 0.0 | 2.0 | 0.7 | 1.1 |
| Defb1 | 3.6 | 0.3 | 0.0 | 0.0 | 0.4 | 0.0 | 0.0 | 0.0 | 0.0 |
| Defb30 | 3.6 | 0.0 | 0.0 | 0.0 | 0.0 | 0.0 | 0.0 | 0.0 | 1.1 |
| Defb35 | 3.6 | 0.0 | 0.1 | 0.0 | 0.0 | 0.0 | 0.0 | 0.1 | 0.0 |
| Fga | 3.6 | 0.0 | 0.2 | 0.0 | 0.1 | 0.0 | 0.0 | 1.3 | 3.2 |
| Gbp1 | 3.6 | 40.2 | 58.0 | 11.1 | 30.9 | 2.8 | 27.5 | 66.3 | 0.0 |
| Pglyrp3 | 3.6 | 0.0 | 0.1 | 0.0 | 0.0 | 0.0 | 0.0 | 0.1 | 0.0 |
| Pglyrp4 | 3.6 | 1.2 | 0.4 | 0.0 | 0.1 | 0.0 | 0.1 | 0.1 | 1.1 |
| Pomc | 3.6 | 13.2 | 4.2 | 2.6 | 4.8 | 0.8 | 3.4 | 5.6 | 9.6 |
| Ppbp | 3.6 | 0.0 | 0.8 | 0.0 | 0.1 | 0.0 | 0.0 | 0.5 | 0.0 |
| Prf1 | 3.6 | 3.9 | 75.9 | 5.7 | 46.2 | 11.6 | 13.4 | 74.7 | 1.1 |
| Rarres2 | 3.6 | 2.4 | 0.3 | 0.0 | 0.1 | 0.0 | 0.3 | 0.6 | 5.3 |
| Retn | 3.6 | 0.6 | 0.1 | 0.0 | 0.9 | 0.0 | 0.3 | 0.1 | 16.0 |
| Ang2 | 2.4 | 1.8 | 2.2 | 0.1 | 1.3 | 0.1 | 0.3 | 3.2 | 0.0 |
| Bpifa1 | 2.4 | 0.0 | 0.1 | 0.0 | 0.0 | 0.0 | 0.1 | 0.1 | 3.2 |
| Bpifb4 | 2.4 | 0.0 | 0.3 | 0.0 | 0.1 | 0.0 | 0.0 | 0.3 | 4.3 |
| Camp | 2.4 | 0.3 | 0.1 | 0.0 | 0.8 | 0.0 | 0.0 | 0.0 | 1.1 |
| Ccl17 | 2.4 | 1.8 | 0.2 | 0.0 | 0.2 | 0.0 | 0.7 | 0.1 | 26.6 |
| Ccl22 | 2.4 | 87.7 | 4.8 | 0.0 | 1.2 | 1.3 | 2.0 | 1.6 | 1.1 |
| Ccl24 | 2.4 | 11.7 | 0.1 | 0.0 | 0.3 | 0.6 | 0.0 | 0.1 | 1.1 |
| Serpind1 | 2.4 | 1.8 | 2.2 | 0.0 | 1.6 | 1.4 | 0.0 | 2.1 | 6.4 |
| Adm | 1.2 | 4.2 | 0.7 | 0.0 | 3.7 | 0.0 | 0.9 | 0.3 | 9.6 |
| Bpifa2 | 1.2 | 0.0 | 0.2 | 0.0 | 0.0 | 0.0 | 0.0 | 0.1 | 0.0 |
| Bst2 | 1.2 | 76.0 | 58.0 | 21.5 | 37.8 | 7.4 | 33.4 | 55.1 | 6.4 |
| Cxcl13 | 1.2 | 0.0 | 37.5 | 0.0 | 18.4 | 0.1 | 0.1 | 13.3 | 0.0 |
| Cxcl3 | 1.2 | 8.4 | 0.7 | 0.0 | 3.4 | 0.0 | 0.8 | 0.5 | 0.0 |
| Cxcl9 | 1.2 | 11.7 | 0.3 | 0.0 | 1.3 | 0.0 | 2.2 | 0.4 | 0.0 |
| Defb2 | 1.2 | 0.0 | 0.0 | 0.0 | 0.1 | 0.1 | 0.0 | 0.0 | 2.1 |
| Defb3 | 1.2 | 0.0 | 0.0 | 0.0 | 0.0 | 0.0 | 0.0 | 0.0 | 0.0 |
| Defb5 | 1.2 | 0.0 | 0.0 | 0.0 | 0.0 | 0.0 | 0.0 | 0.0 | 1.1 |
| Defb11 | 1.2 | 0.0 | 0.0 | 0.0 | 0.0 | 0.0 | 0.0 | 0.0 | 1.1 |
| Defb13 | 1.2 | 0.0 | 0.0 | 0.0 | 0.0 | 0.0 | 0.0 | 0.0 | 0.0 |
| Defb15 | 1.2 | 0.0 | 0.0 | 0.0 | 0.0 | 0.0 | 0.0 | 0.0 | 0.0 |
| Defb28 | 1.2 | 0.0 | 0.0 | 0.0 | 0.0 | 0.0 | 0.0 | 0.0 | 0.0 |
| Defb29 | 1.2 | 0.3 | 0.0 | 0.0 | 0.1 | 0.0 | 0.0 | 0.1 | 0.0 |
| F2 | 1.2 | 0.0 | 0.4 | 0.0 | 0.2 | 0.0 | 0.0 | 0.7 | 0.0 |
| Flg2 | 1.2 | 61.6 | 0.6 | 0.1 | 0.6 | 0.3 | 0.0 | 0.6 | 2.1 |
| Galp | 1.2 | 0.0 | 0.2 | 0.0 | 0.0 | 0.0 | 0.0 | 0.0 | 0.0 |
| Gbp2 | 1.2 | 37.2 | 80.4 | 25.6 | 49.7 | 5.4 | 54.7 | 78.4 | 3.2 |
| Gpr15 | 1.2 | 0.0 | 0.0 | 0.0 | 0.0 | 0.0 | 0.0 | 0.0 | 0.0 |
| Hp | 1.2 | 0.3 | 0.4 | 0.0 | 0.4 | 0.0 | 0.0 | 1.8 | 4.3 |
| Ifnl3 | 1.2 | 0.3 | 0.1 | 0.0 | 0.0 | 0.0 | 0.0 | 0.6 | 0.0 |
| Klk5 | 1.2 | 0.0 | 0.2 | 0.0 | 0.1 | 0.0 | 0.0 | 0.1 | 0.0 |
| Leap2 | 1.2 | 1.2 | 2.6 | 3.2 | 3.4 | 0.5 | 4.1 | 2.4 | 2.1 |
| Ltf | 1.2 | 0.0 | 1.0 | 0.0 | 0.2 | 0.0 | 0.1 | 0.7 | 1.1 |
| Lyz1 | 1.2 | 100.0 | 4.3 | 1.0 | 10.8 | 0.3 | 2.6 | 2.1 | 0.0 |
| Nts | 1.2 | 0.0 | 0.1 | 0.0 | 0.0 | 0.0 | 0.0 | 0.1 | 30.9 |
| Pla2g2a | 1.2 | 0.0 | 0.4 | 0.0 | 0.0 | 0.0 | 0.2 | 0.0 | 1.1 |
| Prg2 | 1.2 | 0.0 | 17.8 | 0.0 | 0.4 | 0.0 | 0.0 | 15.6 | 0.0 |
| Rnase6 | 1.2 | 68.8 | 1.8 | 0.3 | 10.2 | 0.1 | 0.2 | 4.2 | 2.1 |
| S100a7a | 1.2 | 0.0 | 0.8 | 0.0 | 0.1 | 0.0 | 0.1 | 0.3 | 1.1 |
| S100a9 | 1.2 | 26.1 | 2.8 | 0.7 | 7.4 | 1.1 | 3.6 | 1.5 | 1.1 |
| Spag11b | 1.2 | 0.0 | 0.1 | 0.0 | 0.1 | 0.0 | 0.0 | 0.0 | 1.1 |
| Xcl1 | 1.2 | 0.3 | 14.2 | 0.5 | 11.0 | 6.9 | 4.6 | 13.4 | 0.0 |
| Zg16 | 1.2 | 4.8 | 2.1 | 0.2 | 0.8 | 0.1 | 0.4 | 1.4 | 0.0 |
| **Average**  **±SD** | **11.1±16.9** | **16.9±27.8** | **14.0±27.0** | **5.7±17.4** | **10.0±21.0** | **3.8±12.5** | **7.7±19.6** | **13.2±26.3** | **17.2±26.5** |

**Supplementary Table 4.** The ON state frequencies (%) of immune process genes in our single-neuron transcriptomic data and in online datasets obtained from immune cells and hippocampal neurons (for references see Materials and Methods in the main text). The genes are arranged in descending order based on our results. Averages are shown in the last line. (Abbreviations: PFC, prefrontal cortex; CRC, colorectal cancer; UMI, unique molecular identifier; BAL, bronchoalveolar lavage; LC, liver cancer; HPC, hippocampus.)

| **Dataset** | **Own data** | **GSE**  **100337** | **GSE**  **108989** | | **GSE**  **119373** | | **GSE**  **120575** | **GSE**  **124675** | **GSE**  **126030** | **GSE**  **98638** | **GSE**  **75386** | |
| --- | --- | --- | --- | --- | --- | --- | --- | --- | --- | --- | --- | --- |
| **Method** | **HiSeq2500/ NextSeq500** | **Smart-**  **seq2** | **Smart-seq2** | | **Chromium** | | **Smart-seq2** | **SMARTer (UMI)** | **Chromium** | **Tang & Smart-**  **seq2** | **NextSeq 500** | |
| **Species** | **Mouse** | **Human** | **Human** | | **Human** | | **Human** | **Human** | **Human** | **Human** | **Mouse** | |
| **Cell type** | **PFC neurons** | **Mono-phago-**  **cytes** | **T cells (CRC)** | | **CD4+ CD25+ T cells** | | **CD45 cells** | **T cells (BAL)** | **T cells** | **T cells**  **(LC)** | **HPC neurons** | |
| **Gene** | **ON state frequency (%)** | | | | | | | | | | | |
| Canx | 77.4 | 89.8 | | 71.4 | | 14.1 | 48.2 | 4.7 | 25.7 | 70.8 | | 90.4 |
| Prkcb | 73.8 | 48.3 | | 47.0 | | 12.4 | 36.6 | 6.8 | 10.3 | 48.4 | | 80.9 |
| Rac1 | 66.7 | 79.0 | | 49.6 | | 26.9 | 22.5 | 8.8 | 43.6 | 49.5 | | 85.1 |
| Cd81 | 65.5 | 31.8 | | 18.8 | | 7.0 | 17.8 | 6.4 | 6.2 | 22.3 | | 87.2 |
| Calr | 61.9 | 98.8 | | 86.4 | | 26.4 | 89.4 | 8.0 | 49.3 | 85.0 | | 87.2 |
| Itpr1 | 59.5 | 35.7 | | 43.6 | | 2.8 | 29.3 | 2.8 | 4.0 | 41.9 | | 71.3 |
| Mapk1 | 58.3 | 24.6 | | 42.1 | | 7.4 | 15.0 | 3.6 | 13.4 | 44.9 | | 79.8 |
| Ticam1 | 58.3 | 29.7 | | 15.6 | | 1.9 | 11.4 | 0.5 | 6.0 | 15.8 | | 12.8 |
| Fos | 53.6 | 99.7 | | 85.4 | | 5.9 | 69.3 | 2.1 | 5.1 | 35.4 | | 78.7 |
| Pak1 | 53.6 | 74.2 | | 14.5 | | 1.2 | 11.4 | 0.9 | 3.1 | 12.2 | | 80.9 |
| Creb1 | 52.4 | 69.1 | | 51.8 | | 12.6 | 24.9 | 2.8 | 14.0 | 50.8 | | 53.2 |
| Jun | 51.2 | 80.2 | | 72.2 | | 10.3 | 52.2 | 14.9 | 12.6 | 45.5 | | 63.8 |
| Mapk14 | 48.8 | 15.0 | | 27.4 | | 6.4 | 10.3 | 0.8 | 5.4 | 28.7 | | 62.8 |
| Mapk9 | 48.8 | 10.5 | | 11.6 | | 2.4 | 6.6 | 1.3 | 3.4 | 13.1 | | 70.2 |
| Jak1 | 46.4 | 76.3 | | 90.7 | | 41.9 | 58.3 | 9.4 | 30.7 | 90.5 | | 79.8 |
| Ptk2b | 46.4 | 49.8 | | 68.1 | | 7.9 | 50.3 | 6.3 | 7.1 | 68.9 | | 69.1 |
| Grb2 | 44.0 | 96.1 | | 75.5 | | 18.1 | 58.3 | 14.2 | 20.8 | 72.4 | | 67.0 |
| Map2k1 | 41.7 | 14.1 | | 34.7 | | 9.5 | 10.6 | 2.6 | 18.1 | 32.2 | | 70.2 |
| B2m | 40.5 | 100.0 | | 100.0 | | 100.0 | 99.9 | 99.7 | 100.0 | 100.0 | | 62.8 |
| Sos1 | 40.5 | 20.1 | | 33.2 | | 5.7 | 18.5 | 1.0 | 7.0 | 41.9 | | 61.7 |
| Lgmn | 39.3 | 88.6 | | 11.7 | | 1.2 | 8.2 | 1.0 | 1.9 | 3.7 | | 78.7 |
| Map2k2 | 35.7 | 23.7 | | 32.9 | | 8.3 | 26.3 | 6.1 | 22.2 | 31.7 | | 60.6 |
| Pdpk1 | 35.7 | 32.7 | | 24.3 | | 4.4 | 20.5 | 2.5 | 6.4 | 25.9 | | 71.3 |
| Map3k7 | 33.3 | 25.5 | | 37.3 | | 6.2 | 15.4 | 2.3 | 6.7 | 37.9 | | 54.3 |
| Ctss | 31.0 | 99.4 | | 66.6 | | 24.0 | 42.0 | 4.2 | 18.4 | 67.2 | | 59.6 |
| Jak2 | 29.8 | 55.3 | | 24.2 | | 1.6 | 14.8 | 0.7 | 2.5 | 27.6 | | 50.0 |
| Map3k1 | 28.6 | 65.5 | | 38.0 | | 15.2 | 22.1 | 2.3 | 5.7 | 37.7 | | 34.0 |
| Tpp2 | 28.6 | 59.2 | | 59.6 | | 12.2 | 38.1 | 2.4 | 7.6 | 60.9 | | 69.1 |
| Traf3 | 28.6 | 20.1 | | 35.3 | | 4.3 | 20.9 | 0.9 | 10.0 | 27.5 | | 57.4 |
| Mapk3 | 27.4 | 11.4 | | 9.4 | | 4.1 | 5.5 | 1.9 | 6.3 | 10.6 | | 55.3 |
| Pdia3 | 27.4 | 88.3 | | 88.8 | | 30.3 | 60.2 | 7.3 | 40.9 | 83.0 | | 70.2 |
| Raf1 | 26.2 | 35.7 | | 56.5 | | 10.6 | 29.7 | 3.1 | 13.5 | 54.6 | | 66.0 |
| Malt1 | 25.0 | 19.8 | | 42.4 | | 12.4 | 16.1 | 3.1 | 10.3 | 32.5 | | 28.7 |
| Nfkbia | 25.0 | 80.2 | | 90.5 | | 22.1 | 65.3 | 15.7 | 53.4 | 61.6 | | 57.4 |
| Akt1 | 21.4 | 18.9 | | 16.6 | | 4.2 | 11.6 | 3.0 | 4.9 | 16.0 | | 67.0 |
| Nfatc2 | 20.2 | 30.9 | | 50.1 | | 6.7 | 29.0 | 7.1 | 9.0 | 52.4 | | 46.8 |
| Fcer1g | 19.0 | 87.7 | | 5.0 | | 5.5 | 13.4 | 1.9 | 4.5 | 3.0 | | 54.3 |
| Map2k3 | 16.7 | 38.4 | | 53.3 | | 7.2 | 33.7 | 0.9 | 17.2 | 45.8 | | 45.7 |
| Map2k7 | 16.7 | 9.3 | | 14.2 | | 6.6 | 9.3 | 3.0 | 11.4 | 17.3 | | 53.2 |
| Tyk2 | 16.7 | 55.0 | | 40.1 | | 4.7 | 28.6 | 3.1 | 4.9 | 41.2 | | 30.9 |
| Cd247 | 15.5 | 0.9 | | 83.1 | | 34.0 | 41.8 | 8.5 | 37.0 | 81.7 | | 1.1 |
| Cd79a | 15.5 | 2.7 | | 10.1 | | 2.3 | 13.4 | 0.0 | 0.7 | 5.1 | | 3.2 |
| Rasgrp3 | 15.5 | 18.3 | | 6.0 | | 1.3 | 8.8 | 2.2 | 3.2 | 6.2 | | 17.0 |
| Blnk | 14.3 | 26.7 | | 0.6 | | 0.1 | 9.9 | 0.0 | 0.1 | 0.2 | | 34.0 |
| Stat6 | 14.3 | 71.5 | | 50.0 | | 10.8 | 41.6 | 25.3 | 14.0 | 56.1 | | 11.7 |
| Plcg2 | 13.1 | 24.6 | | 14.6 | | 3.5 | 15.3 | 1.5 | 0.6 | 9.0 | | 14.9 |
| Traf2 | 13.1 | 20.7 | | 24.7 | | 1.9 | 14.6 | 1.6 | 8.1 | 21.6 | | 24.5 |
| Ccr7 | 11.9 | 3.3 | | 69.5 | | 19.7 | 19.2 | 1.5 | 38.4 | 40.3 | | 3.2 |
| Cr2 | 11.9 | 0.9 | | 1.1 | | 0.2 | 4.5 | 0.1 | 0.1 | 0.5 | | 6.4 |
| Stat1 | 11.9 | 79.9 | | 82.9 | | 16.8 | 60.0 | 3.5 | 43.8 | 86.8 | | 40.4 |
| Il2 | 10.7 | 0.6 | | 4.3 | | 0.4 | 0.6 | 0.2 | 3.2 | 1.6 | | 17.0 |
| Il4ra | 10.7 | 27.3 | | 40.9 | | 6.9 | 25.3 | 2.5 | 25.0 | 32.6 | | 16.0 |
| Lyn | 10.7 | 66.4 | | 6.5 | | 0.3 | 12.1 | 0.4 | 2.4 | 7.7 | | 19.1 |
| Aicda | 9.5 | 72.1 | | 0.3 | | 0.2 | 0.4 | 0.3 | 0.1 | 0.2 | | 2.1 |
| Bcl10 | 9.5 | 54.4 | | 33.7 | | 3.8 | 27.9 | 3.3 | 7.9 | 30.3 | | 46.8 |
| Cd19 | 9.5 | 2.1 | | 0.7 | | 0.0 | 8.2 | 0.0 | 0.1 | 0.3 | | 0.0 |
| Cd28 | 9.5 | 29.4 | | 69.2 | | 13.5 | 29.9 | 0.5 | 14.3 | 73.2 | | 4.3 |
| Cd74 | 9.5 | 100.0 | | 98.6 | | 46.1 | 93.9 | 35.1 | 56.9 | 94.8 | | 9.6 |
| Inpp5d | 9.5 | 75.1 | | 75.9 | | 18.5 | 54.5 | 6.7 | 10.6 | 79.6 | | 25.5 |
| Ptpn6 | 9.5 | 82.6 | | 67.2 | | 15.9 | 44.3 | 4.9 | 21.5 | 67.0 | | 18.1 |
| Tap2 | 9.5 | 58.9 | | 32.3 | | 6.7 | 29.8 | 0.8 | 15.0 | 47.6 | | 8.5 |
| Tapbp | 9.5 | 100.0 | | 95.2 | | 25.0 | 87.1 | 22.6 | 46.9 | 95.9 | | 50.0 |
| Traf6 | 9.5 | 63.4 | | 23.4 | | 1.0 | 9.4 | 1.3 | 2.8 | 24.0 | | 34.0 |
| Tap1 | 8.3 | 65.2 | | 92.7 | | 19.2 | 63.6 | 8.2 | 46.3 | 93.5 | | 9.6 |
| Tnfrsf13c | 8.3 | 0.9 | | 0.4 | | 1.5 | 0.6 | 3.6 | 2.4 | 0.2 | | 1.1 |
| Vav1 | 8.3 | 42.0 | | 28.2 | | 3.7 | 26.6 | 3.1 | 6.7 | 27.7 | | 13.8 |
| Cd14 | 7.1 | 47.4 | | 0.7 | | 0.0 | 7.9 | 0.5 | 0.2 | 0.8 | | 30.9 |
| Il12rb2 | 7.1 | 4.8 | | 31.3 | | 1.1 | 7.4 | 0.6 | 3.2 | 26.6 | | 25.5 |
| Il7r | 7.1 | 4.8 | | 90.1 | | 42.5 | 33.6 | 11.7 | 40.3 | 85.5 | | 8.5 |
| Itgal | 7.1 | 82.9 | | 81.6 | | 13.7 | 59.1 | 8.2 | 15.5 | 86.1 | | 6.4 |
| Jak3 | 7.1 | 92.8 | | 86.1 | | 9.9 | 68.1 | 12.9 | 15.8 | 78.5 | | 8.5 |
| Stat5a | 7.1 | 33.9 | | 52.1 | | 4.7 | 23.6 | 1.8 | 14.5 | 41.1 | | 14.9 |
| Card11 | 6.0 | 13.8 | | 33.5 | | 2.4 | 21.3 | 2.3 | 6.2 | 35.8 | | 4.3 |
| Cd22 | 6.0 | 12.3 | | 0.3 | | 0.4 | 8.3 | 1.1 | 0.8 | 0.2 | | 1.1 |
| Cd72 | 6.0 | 46.5 | | 9.4 | | 0.5 | 14.0 | 0.4 | 0.8 | 11.0 | | 9.6 |
| H2-DMa | 6.0 | 99.1 | | 44.8 | | 8.5 | 42.2 | 3.6 | 5.4 | 36.5 | | 37.2 |
| Il12b | 6.0 | 7.5 | | 0.3 | | 0.0 | 0.1 | 0.1 | 0.0 | 0.1 | | 0.0 |
| Map3k14 | 6.0 | 28.8 | | 24.9 | | 2.9 | 17.4 | 1.5 | 6.0 | 27.5 | | 9.6 |
| C3 | 4.8 | 9.3 | | 1.2 | | 0.0 | 2.5 | 0.6 | 0.3 | 2.2 | | 2.1 |
| Cd86 | 4.8 | 75.4 | | 4.5 | | 0.0 | 9.7 | 0.3 | 0.4 | 3.1 | | 7.4 |
| Fasl | 4.8 | 0.6 | | 28.7 | | 0.1 | 13.7 | 3.5 | 3.2 | 33.3 | | 2.1 |
| Fcgr2b | 4.8 | 57.1 | | 1.5 | | 0.0 | 9.4 | 0.2 | 0.1 | 2.5 | | 12.8 |
| Lat | 4.8 | 3.3 | | 89.3 | | 39.7 | 40.3 | 2.1 | 8.2 | 89.0 | | 6.4 |
| Map3k8 | 4.8 | 61.0 | | 38.8 | | 0.5 | 28.8 | 0.9 | 9.0 | 22.0 | | 28.7 |
| Rag2 | 4.8 | 0.0 | | 0.2 | | 0.0 | 0.2 | 0.0 | 0.0 | 0.1 | | 0.0 |
| Syk | 4.8 | 90.4 | | 1.0 | | 0.2 | 13.8 | 0.3 | 0.8 | 0.6 | | 17.0 |
| Tlr4 | 4.8 | 39.6 | | 0.5 | | 0.0 | 4.5 | 0.0 | 0.1 | 0.6 | | 1.1 |
| Btk | 3.6 | 43.8 | | 0.6 | | 0.1 | 9.8 | 0.1 | 0.9 | 0.8 | | 4.3 |
| Cd3d | 3.6 | 0.3 | | 99.8 | | 76.2 | 60.7 | 51.8 | 74.6 | 98.8 | | 1.1 |
| Ifng | 3.6 | 0.6 | | 42.0 | | 0.2 | 25.4 | 2.3 | 17.1 | 26.6 | | 0.0 |
| Il7 | 3.6 | 2.1 | | 8.5 | | 2.1 | 3.4 | 0.1 | 0.4 | 8.4 | | 5.3 |
| Lck | 3.6 | 0.3 | | 97.3 | | 52.9 | 58.0 | 34.1 | 48.8 | 94.4 | | 12.8 |
| Prf1 | 3.6 | 3.9 | | 75.9 | | 5.7 | 46.2 | 11.6 | 13.4 | 74.7 | | 1.1 |
| Prkcq | 3.6 | 1.2 | | 64.8 | | 7.7 | 26.1 | 3.5 | 12.7 | 66.0 | | 11.7 |
| Rag1 | 3.6 | 0.3 | | 0.5 | | 0.0 | 0.3 | 0.0 | 0.1 | 0.5 | | 1.1 |
| Stat4 | 3.6 | 9.9 | | 85.5 | | 7.0 | 49.2 | 6.7 | 14.8 | 85.9 | | 1.1 |
| Tbx21 | 3.6 | 0.0 | | 21.0 | | 0.8 | 7.4 | 2.9 | 8.3 | 26.8 | | 1.1 |
| Ticam2 | 3.6 | 10.2 | | 2.8 | | 0.0 | 3.0 | 0.0 | 0.0 | 2.6 | | 4.3 |
| Tnfsf13b | 3.6 | 66.4 | | 12.8 | | 2.5 | 10.1 | 7.1 | 4.3 | 13.1 | | 5.3 |
| Cd3e | 2.4 | 1.2 | | 97.5 | | 62.0 | 65.8 | 40.6 | 56.8 | 96.9 | | 1.1 |
| Cd4 | 2.4 | 93.7 | | 66.7 | | 17.4 | 28.3 | 2.8 | 8.6 | 64.7 | | 6.4 |
| Gzma | 2.4 | 0.0 | | 75.4 | | 5.0 | 44.3 | 38.1 | 10.0 | 70.3 | | 0.0 |
| Gzmb | 2.4 | 1.2 | | 61.6 | | 0.3 | 31.5 | 55.5 | 29.0 | 36.7 | | 0.0 |
| H2-Oa | 2.4 | 86.8 | | 5.7 | | 0.5 | 13.2 | 0.4 | 0.6 | 4.4 | | 2.1 |
| H2-Ob | 2.4 | 30.3 | | 1.3 | | 0.3 | 6.4 | 0.0 | 0.6 | 0.9 | | 0.0 |
| Il2rb | 2.4 | 0.0 | | 79.3 | | 11.3 | 50.3 | 10.0 | 16.8 | 73.5 | | 0.0 |
| Plcg1 | 2.4 | 2.1 | | 28.7 | | 5.1 | 22.9 | 2.8 | 2.5 | 37.3 | | 53.2 |
| Tnfrsf13b | 2.4 | 3.3 | | 4.6 | | 0.7 | 2.7 | 0.0 | 0.1 | 5.2 | | 2.1 |
| Tnfrsf17 | 2.4 | 0.0 | | 0.2 | | 0.0 | 1.9 | 0.3 | 0.0 | 0.3 | | 2.1 |
| Cd3g | 1.2 | 56.5 | | 94.3 | | 59.2 | 68.5 | 39.6 | 32.3 | 92.6 | | 0.0 |
| Cd40 | 1.2 | 34.5 | | 4.0 | | 0.8 | 11.1 | 0.1 | 2.4 | 2.9 | | 1.1 |
| Cd40lg | 1.2 | 1.5 | | 39.0 | | 7.1 | 7.4 | 4.2 | 12.1 | 45.8 | | 0.0 |
| Cd79b | 1.2 | 3.0 | | 27.5 | | 10.4 | 17.2 | 1.5 | 4.8 | 28.7 | | 2.1 |
| Cd80 | 1.2 | 12.0 | | 9.9 | | 0.1 | 5.0 | 0.2 | 0.3 | 5.0 | | 0.0 |
| Cd8a | 1.2 | 7.5 | | 61.6 | | 0.0 | 43.2 | 22.4 | 16.0 | 46.5 | | 1.1 |
| Ifi30 | 1.2 | 97.9 | | 14.2 | | 0.3 | 22.8 | 0.1 | 0.7 | 13.9 | | 22.3 |
| Il2ra | 1.2 | 36.9 | | 47.3 | | 25.5 | 8.9 | 2.1 | 25.3 | 28.8 | | 1.1 |
| Il4 | 1.2 | 0.0 | | 0.5 | | 0.0 | 0.2 | 0.0 | 0.9 | 0.9 | | 2.1 |
| Il5 | 1.2 | 0.3 | | 0.4 | | 0.0 | 0.5 | 0.0 | 0.5 | 0.5 | | 0.0 |
| Lta | 1.2 | 0.0 | | 17.3 | | 1.6 | 7.8 | 6.4 | 16.0 | 18.8 | | 2.1 |
| Pax5 | 1.2 | 1.2 | | 0.4 | | 0.1 | 7.0 | 2.9 | 0.2 | 0.9 | | 0.0 |
| Zap70 | 1.2 | 0.3 | | 87.1 | | 18.6 | 53.6 | 4.4 | 14.0 | 88.0 | | 7.4 |
| H2-DMb2 | 0.0 | 97.3 | | 26.2 | | 1.7 | 22.8 | 1.8 | 1.3 | 21.2 | | 5.3 |
| Il12a | 0.0 | 0.0 | | 1.7 | | 0.3 | 0.6 | 0.0 | 0.3 | 1.3 | | 3.2 |
| Il12rb1 | 0.0 | 89.8 | | 45.3 | | 6.8 | 18.8 | 3.4 | 6.6 | 45.1 | | 3.2 |
| Il2rg | 0.0 | 58.0 | | 98.8 | | 48.5 | 77.0 | 35.4 | 49.6 | 97.6 | | 3.2 |
| Pik3ap1 | 0.0 | 46.5 | | 15.4 | | 0.4 | 17.2 | 0.8 | 2.3 | 16.1 | | 8.5 |
| Tcra | 0.0 | 0.0 | | 0.0 | | 0.0 | 0.0 | 0.0 | 0.0 | 0.0 | | 0.0 |
| Tcrb | 0.0 | 0.0 | | 0.0 | | 0.0 | 0.0 | 0.0 | 0.0 | 0.0 | | 0.0 |
| **Average**  **±SD** | **16.4±19.0** | **37.9±34.4** | | **38.7±32.6** | | **10.3±16.2** | **26.2±23.0** | **6.6±13.3** | **13.1±17.1** | **36.6±31.5** | | **26.1±28.5** |
